# Understanding the effect of smoking and drinking behavior on Parkinson's disease risk: a Mendelian randomization study

**DOI:** 10.1038/s41598-021-93105-y

**Published:** 2021-07-07

**Authors:** Carmen Domínguez-Baleón, Jue-Sheng Ong, Clemens R. Scherzer, Miguel E. Rentería, Xianjun Dong

**Affiliations:** 1 Center for Advanced Parkinson Research and Precision Neurology Program, Brigham & Women’s Hospital, Harvard Medical School, Boston, MA USA; 2 Department of Genetics & Computational Biology, QIMR Berghofer Medical Research Institute, Brisbane, QLD Australia; 3 Department of Neurology, Massachusetts General Hospital, Boston, MA USA; 4 Department of Neurology, Brigham and Women’s Hospital, Boston, MA USA; 5 Genomics and Bioinformatics Hub, Brigham and Women’s Hospital, Boston, MA USA

**Keywords:** Computational biology and bioinformatics, Genetics, Risk factors, Parkinson's disease

## Abstract

Previous observational studies have identified correlations between Parkinson’s disease (PD) risk and lifestyle factors. However, whether or not those associations are causal remains unclear. To infer causality between PD risk and smoking or alcohol intake, we conducted a two-sample Mendelian randomization study using genome-wide association study summary statistics from the GWAS & Sequencing Consortium of Alcohol and Nicotine use study (1.2 million participants) and the latest meta-analysis from the International Parkinson’s Disease Genomics Consortium (37,688 PD cases and 18,618 proxy-cases). We performed sensitivity analyses, including testing for pleiotropy with MR-Egger and MR-PRESSO, and multivariable MR modeling to account for the genetic effects of competing substance use traits on PD risk. Our results revealed causal associations of *alcohol intake* (OR 0.79; 95% CI 0.65–0.96; p = 0.021) and *smoking continuation* (which compares *current* vs. *former smokers*) (OR 0.64; 95% CI 0.46–0.89; p = 0.008) with lower PD risk. Multivariable MR analyses showed that the causal association between *drinks per week* and PD is unlikely due to confounding by smoking behavior. Finally, frailty analyses suggested that the causal effects of both *alcohol intake* and *smoking continuation* on PD risk estimated from MR analysis are not explained by the presence of survival bias alone. Our findings support the role of smoking as a protective factor against PD, but only when comparing *current vs. former smokers*. Similarly, increased alcohol intake had a protective effect over PD risk, with the *alcohol dehydrogenase 1B* (*ADH1B*) locus as a potential candidate for further investigation of the mechanisms underlying this association.

## Introduction

Parkinson’s disease (PD) is a complex genetic disease, and growing evidence exists that lifestyle and environmental factors^1^ and numerous genetic variants modulate both its susceptibility^2,3^ and progression^4,5^. Environmental factors such as exposure to pesticides^6,7^ or history of melanoma^8^ have been associated with increased PD risk, whereas alcohol intake^9–11^, smoking^12–14^, caffeine consumption^15,16^, and educational attainment^17^ are associated with reduced risk^1,18^. However, these relationships could be the product of confounding variables or reverse causation^19,20^. The traditional approach to investigating causality is the *randomized controlled trial* (RCT), where participants are randomly allocated to either a control or a treatment group to reduce bias^21^. However, running an RCT may not be possible in some situations due to the ethical and health concerns associated with subjecting participants to exposures that might be detrimental to their health, such as smoking, drinking, or pesticides^22–24^.

**Figure 1:**
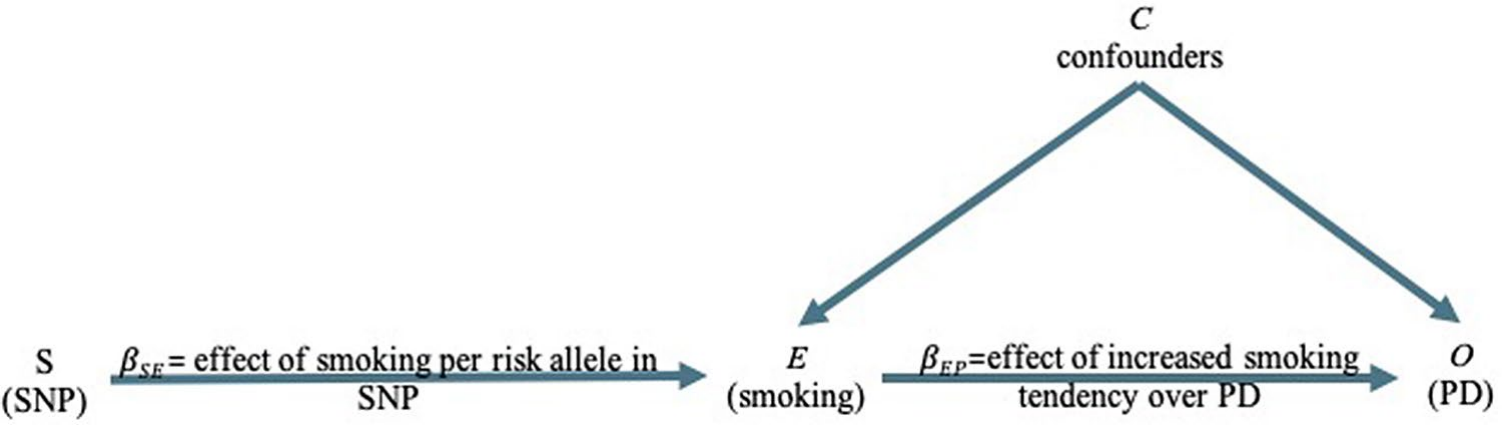
Directed acyclic graph of the effect of smoking on PD using genetic variants (SNPs) as proxies. (Adapted from Lawlor et al.^22^). An SNP (S) is associated with the exposure, for example, smoking (E), and therefore the SNP (S) can be used as an instrument to determine the effect of smoking (*E*) on PD, which is the outcome of interest (*O*). An association between the smoking-risk-increasing SNP (*S*) and elevated risk of PD is consistent with a causal relationship between *E* and *O* if three assumptions are met: (1) The SNP (*S*) must be strongly associated with smoking (*E*); (2) The SNP (*S*) must be independent of any confounding factors (*C*) that may be influencing PD (*O*) and (3) The SNP (S)’s association with PD (*O*) is explained only via smoking (*E*). SNP, single nucleotide polymorphism.

Mendelian randomization (MR) is an exciting alternative methodology that overcomes these difficulties using genetic variants (e.g., single nucleotide polymorphisms, or SNPs) as instruments to infer causality between exposure variables and health outcomes in a way that is independent of confounding factors. The method takes advantage of the fact that genetic variants are randomly distributed in the population just as participants would randomly be allocated in an RCT^20,22,24,25^. A *two-sample MR* experimental design requires summary statistics from two independent genome-wide association studies (GWAS) for the exposure and outcome variables. It examines whether the SNPs (*S*) that influence the exposure (*E*) also affect the outcome (*O*), but not the other way around. This way, MR facilitates the inference of a direct causal relationship between the exposure and the outcome (see Fig. 1)^22,23^.

The inverse association of PD risk with smoking and alcohol intake has been consistently reported in the literature^1,9–14,18^. A recent meta-analysis across 61 case–control and eight cohort studies by Li et al.^13^ estimated pooled relative risk (RR) of a 0.59 (95% CI 0.56–0.62) for PD in smokers. Furthermore, another meta-analysis of 32 studies by Zhang et al.^10^ estimated a RR of 0.78 (95% CI 0.67–0.92) when comparing participants with the highest versus the lowest levels of alcohol intake while also controlling for the effects of smoking and caffeine intake. Notably, between-study heterogeneity has been noted, which implies that studies in much larger and homogeneous samples might still be needed to confirm the causal nature of this association.

Mendelian randomization has recently been applied to infer causality between PD risk and environmental and lifestyle variables. Notably, Nalls et al*.*^26^ reported little evidence for a causal relationship between current tobacco use and PD risk, despite finding a significant inverse genetic correlation between the two traits, estimated using LD score regression. Also, Noyce et al.^27^ conducted a comprehensive investigation of 401 exposure traits, including smoking and drinking, and reported negative causal associations. However, a more detailed examination of the possible horizontal pleiotropy (i.e., a situation where candidate SNP instruments might be associated with the outcome through pathways independent of the exposure of interest) is required to disentangle the interplay between smoking behavior, alcohol intake, and PD risk.

Here we leverage the availability of updated GWAS summary statistics from two recent studies by the GWAS & Sequencing Consortium of Alcohol and Nicotine use (GSCAN)^28^ and the International Parkinson’s Disease Genomics Consortium^26^ (IPDGC) to expand on previous MR investigations. We specifically examine the causal effects of five lifestyle variables (number of alcoholic drinks per week, smoking initiation, heaviness, continuation, and age at initiation) on PD risk. Notably, given that smoking and alcohol intake are both phenotypically and genetically correlated, we performed multivariable MR analysis to control for horizontal pleiotropy on competing substance use factors, thus providing a better adjusted and more robust inference model. Lastly, we performed frailty analyses to test if survival bias was responsible for the MR estimates obtained in this study.

## Results

### Overview

**Figure 2:**
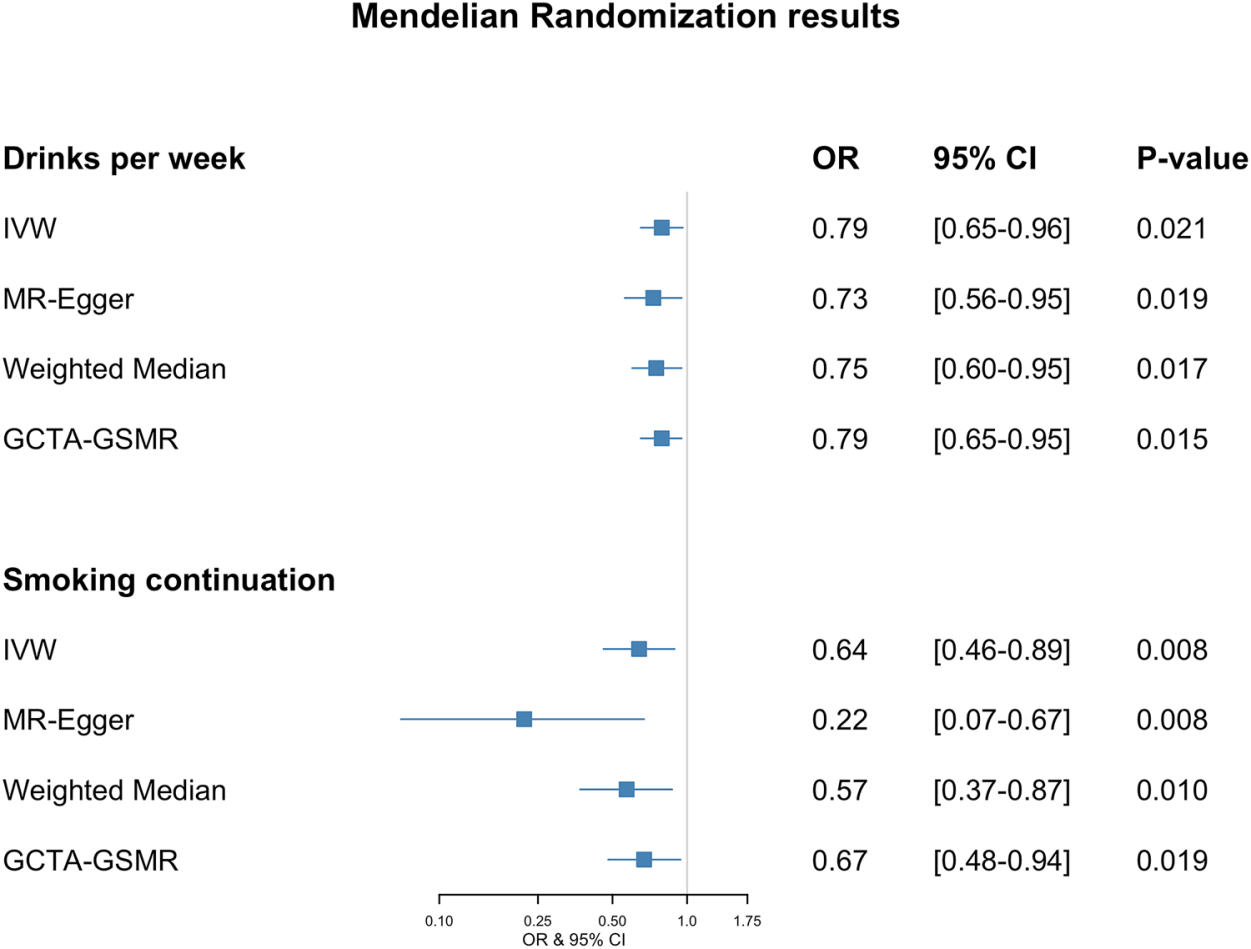
Forest plot showing OR and 95% confidence intervals of MR effect estimates. OR values for *smoking continuation* are expressed in *per doubling of odds* units.

We found strong evidence supporting an inverse (i.e., protective) effect for the *number of drinks per week* and *smoking continuation* (which compares *current vs. former smokers*) traits on PD risk. Nonetheless, there was no clear association between PD risk and *smoking initiation* (*ever vs. never being a regular smoker*), *smoking heaviness* (*number of cigarettes per day*), or the *age at smoking initiation*. The results are summarized in Fig. 2 and Supplementary Fig. S3. Variant data used in this study can be found in Supplementary Tables S1–S5.

### Alcohol intake

**Figure 3:**
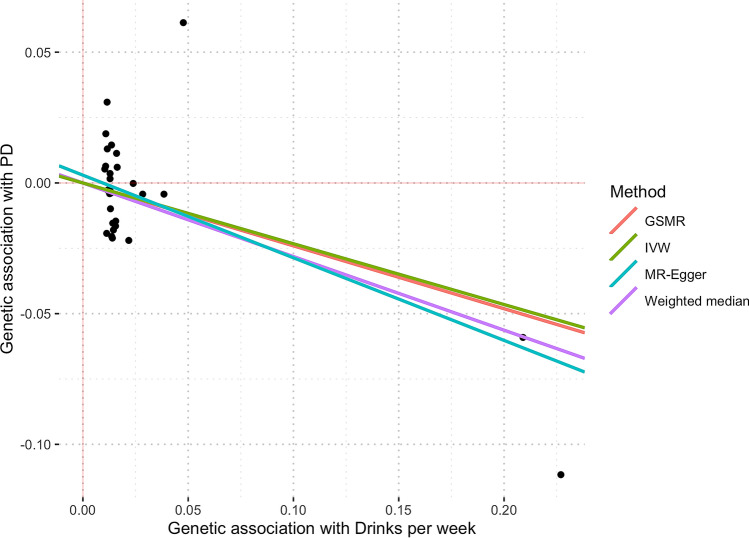
Scatter plot showing MR effect estimates of *drinks per week* over PD. Each SNP-PD association is plotted against SNP-*drinks per week* association, and the corresponding MR estimates for IVW, MR-Egger, Weighted-Median, and GSMR are plotted. The two rightmost variants shown on the plot are rs29001570 and rs1229984, which correspond to the Alcohol Dehydrogenase 5 and Alcohol Dehydrogenase 1B loci.

Horizontal pleiotropy analysis with MR-PRESSO identified two potentially pleiotropic outliers (rs1260326 and rs34121753), which have previously been associated with other traits, such as triglyceride^29^ and serum acid levels^30^ for the former and hematocrit levels for the latter^31^. After excluding outliers, our MR estimates showed that the genetic tendency to have more *drinks per week* is suggested to be causally associated with a reduced PD risk. The OR from the IVW estimate was 0.79 (95% CI 0.65–0.96; p = 0.021) (Fig. 2), with no evidence for horizontal pleiotropy (MR-Egger estimate of 0.73 (95% CI 0.56–0.95; p = 0.019) intercept of 0.003; p = 0.348). Estimates derived from GCTA-GSMR yielded very similar findings: OR was 0.79 (95% CI 0.65–0.95; p = 0.015). There was moderate evidence of heterogeneity of effect estimates between variants (Q statistic = 37.43, I^2^ = 19.85%, p = 0.16). Figure 3 shows all the derived MR estimates from alternative models for the effect of *drinks per week* on PD risk. We did not find evidence for unbalanced pleiotropy via manual inspection in the MR funnel plots (see Supplementary Fig. S1). Notably, the rs1229984 variant in the *alcohol dehydrogenase 1B* (*ADH1B*) locus had the greatest association with *drinks per week*. It is known that individuals with one or two A alleles (i.e., AG or AA genotypes) at rs1229984 are more likely to find drinking unpleasant and thus have a reduced risk for alcoholism^32,33^. After removing rs1229984 and re-running all analyses, the resulting estimate was only slightly attenuated towards the null, and the point estimate remained in the same direction [0.84 (95% CI 0.63–1.11; p = 0.216)], albeit with lower precision. On the other hand, the effect estimate of the rs1229984 single variant on PD risk was OR 0.75 (95% CI 0.59–0.96; p = 0.025).

There was very weak evidence for an association through reverse causality as evaluated in our bidirectional MR analysis. Greater genetic predisposition towards PD did not influence *drinks per week,* with an IVW estimate of 1.006 (95% CI 0.999–1.013; p = 0.083) and the equivalent GSMR estimate of 1.037 (95% CI 1.009–1.066; p = 0.009) per doubling of odds on PD. These findings infer that the association between alcohol drinks/week and PD is likely explained by unidirectional causality where genetically increased alcohol consumption reduces PD risk.

Finally, multivariable MR analysis accounting for the genetic effect of alcohol SNPs on smoking phenotypes found no attenuation of the effect of alcohol on PD risk [i.e., conditional association OR 0.72 (95% CI 0.53–0.98; p = 0.034), per standard deviation increase in the number of drinks per week] when the genetic effect of alcohol SNPs on smoking behavior, obesity and education attainment have been taken into consideration (see “Methods” section).

### Smoking continuation

**Figure 4:**
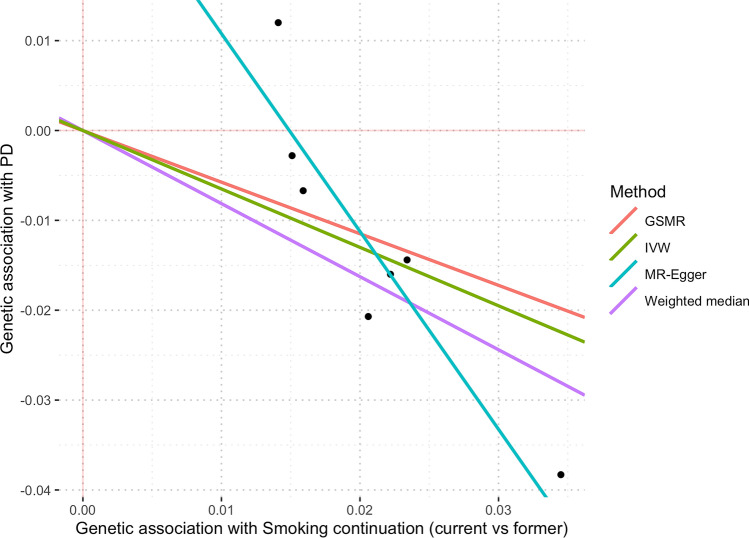
Scatter plot showing MR effect estimates of *smoking continuation* over PD. Each SNP-PD association is plotted against SNP-*smoking continuation* association, and the corresponding MR estimates for IVW, MR-Egger, Weighted-Median and GSMR are plotted.

MR-PRESSO identified a single SNP outlier (rs9607805, known to be associated with neuroticism^34^), which was excluded from the analysis. IVW results showed that current smokers have a lower risk of PD in contrast to former smokers. Based on the IVW estimate, the derived OR on PD per doubling of odds for smoking *continuation* was 0.64 (95% CI 0.46–0.89; p = 0.008) (see Fig. 2). There was weak evidence for unbalanced horizontal pleiotropy biasing the result (MR-Egger estimate was 0.22 (95% CI 0.07–0.67; p = 0.008) per doubling of odds with an intercept (in log(OR)) of 0.033, p = 0.051. No evidence of heterogeneity among variants (Q statistics = 5.48, I^2^ = 0%, p = 0.48) was observed. GSMR analysis results yielded very similar results to IVW, with OR = 0.67 (95% CI 0.48–0.94; p = 0.019) per doubling of odds. Figure 4 shows MR estimates for the effect of *smoking continuation* on PD. Given the existing discrepancies in results from IVW/GSMR and MR-Egger, it is impossible to rule out the possibility that pleiotropic effects may influence the association between *being a current smoker* and PD. Manual inspection of the funnel plot showed asymmetry around the point estimate, which might indicate the presence of horizontal pleiotropy (see Supplementary Fig. S2).

Bidirectionality analysis showed that the effect of predisposition to Parkinson’s disease over *smoking continuation* is very close to the null, with a non-significant p-value and an IVW estimate of 0.997 (95% CI 0.992–1.001; p = 0.14) per doubling of odds. GSMR analysis yielded similar results: 0.96 (95% CI 0.93–0.992; p = 0.015), per doubling of odds of PD on *being a current smoker*. Notably, the multivariable MR OR estimate for smoking *continuation* controlling for the genetic effect on alcohol consumption and other smoking behavior phenotypes could not be meaningfully interpreted due to the low number of SNP instruments, which yields an extensive confidence interval OR 0.66 (95% CI 0.04–9.96; p = 0.76).

### Smoking initiation, heaviness, and age at initiation

The predisposition to *ever being a regular smoker* (as opposed to never initiating smoking) OR per doubling of odds for IVW was 0.90 (95% CI 0.74–1.09; p = 0.308), GSMR 0.89 (95% CI 0.78–0.97, p = 0.112) and MR-Egger 0.39 (95% CI 0.15–1.07; p = 0.068) per doubling of odds with moderate evidence of heterogeneity between variants (Q statistic = 152.78, I^2^ = 44.36%, p = 9.18e-6. *Smoking heaviness*, characterized by the number of cigarettes smoked per day, showed a IVW OR of 1.06 (95% CI 0.88–1.29; p = 0.526), a GSMR estimate of 1.07 (95% CI 0.92–1.23, p = 0.373) and MR-Egger estimate was OR = 1.07 (95% CI 0.75–1.51, p = 0.72) with moderate evidence of heterogeneity (Q statistic = 36.78, I^2^ = 42.9%, p = 0.017). Lastly, *age at smoking initiation* IVW estimate was 0.83 (95% CI 0.53–1.30; p = 0.416), GSMR OR = 0.84 (95% CI 0.52–1.38, p = 0.492) and a MR-Egger estimate of 1.002 (95% CI 0.16–6.29, p = 0.998), showing moderate evidence of heterogeneity (Q statistic = 5.43 I^2^ = 8, p = 0.36) (see Supplementary Fig. S3). Derived MR estimates and corresponding funnel plots for these traits can be found in Supplementary Fig. S5–S10.

### Frailty analysis

**Figure 5:**
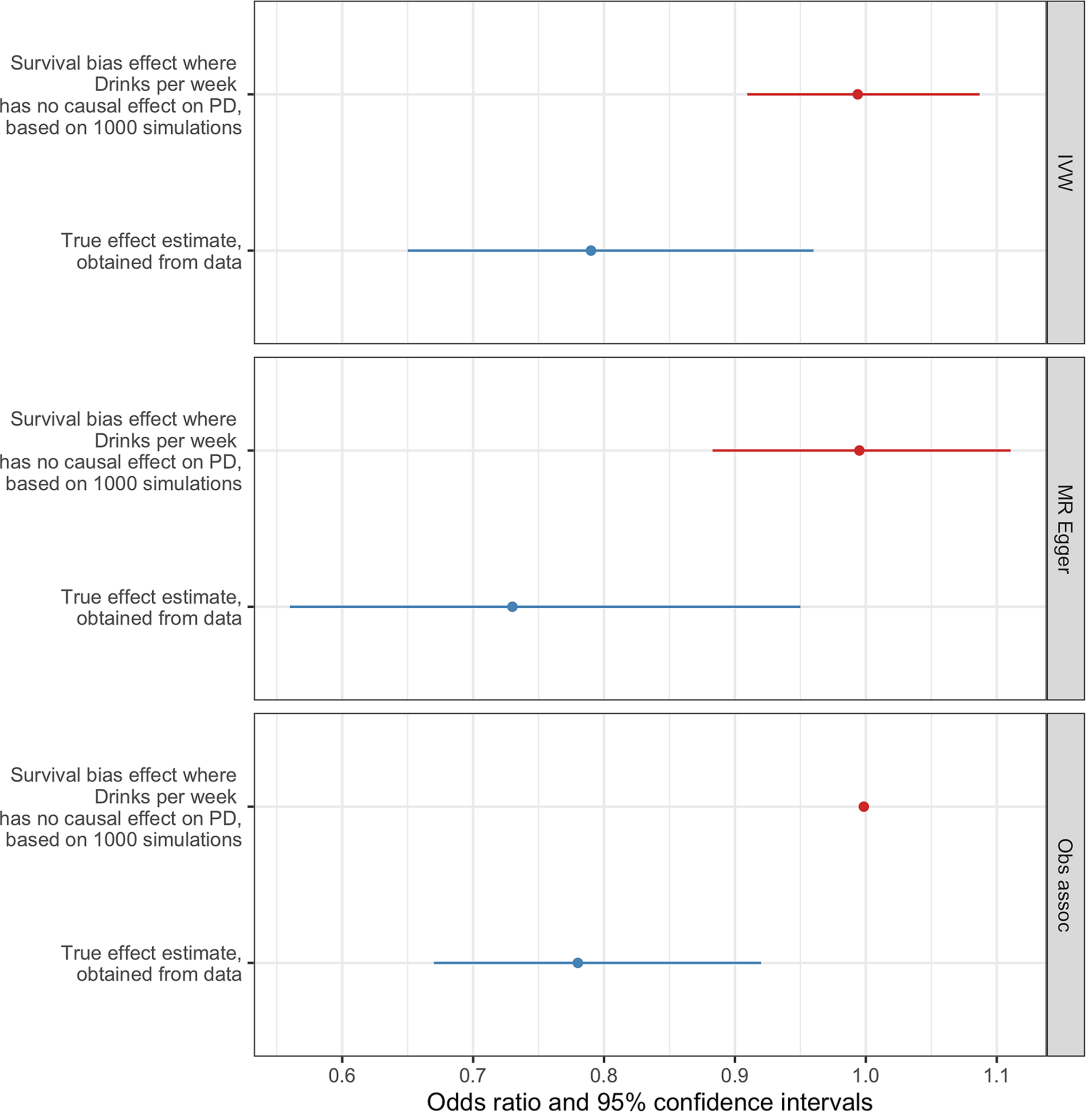
Frailty analysis results for *drinks per week*. The effect estimates obtained from simulations in which the effect *drinks per week* has over PD risk are solely explained by the presence of survival bias (red) are compared to the causal effect estimates between *drinks per week* and PD risk obtained from our MR analysis (blue), for the IVW, MR-Egger and observational approaches.

**Figure 6:**
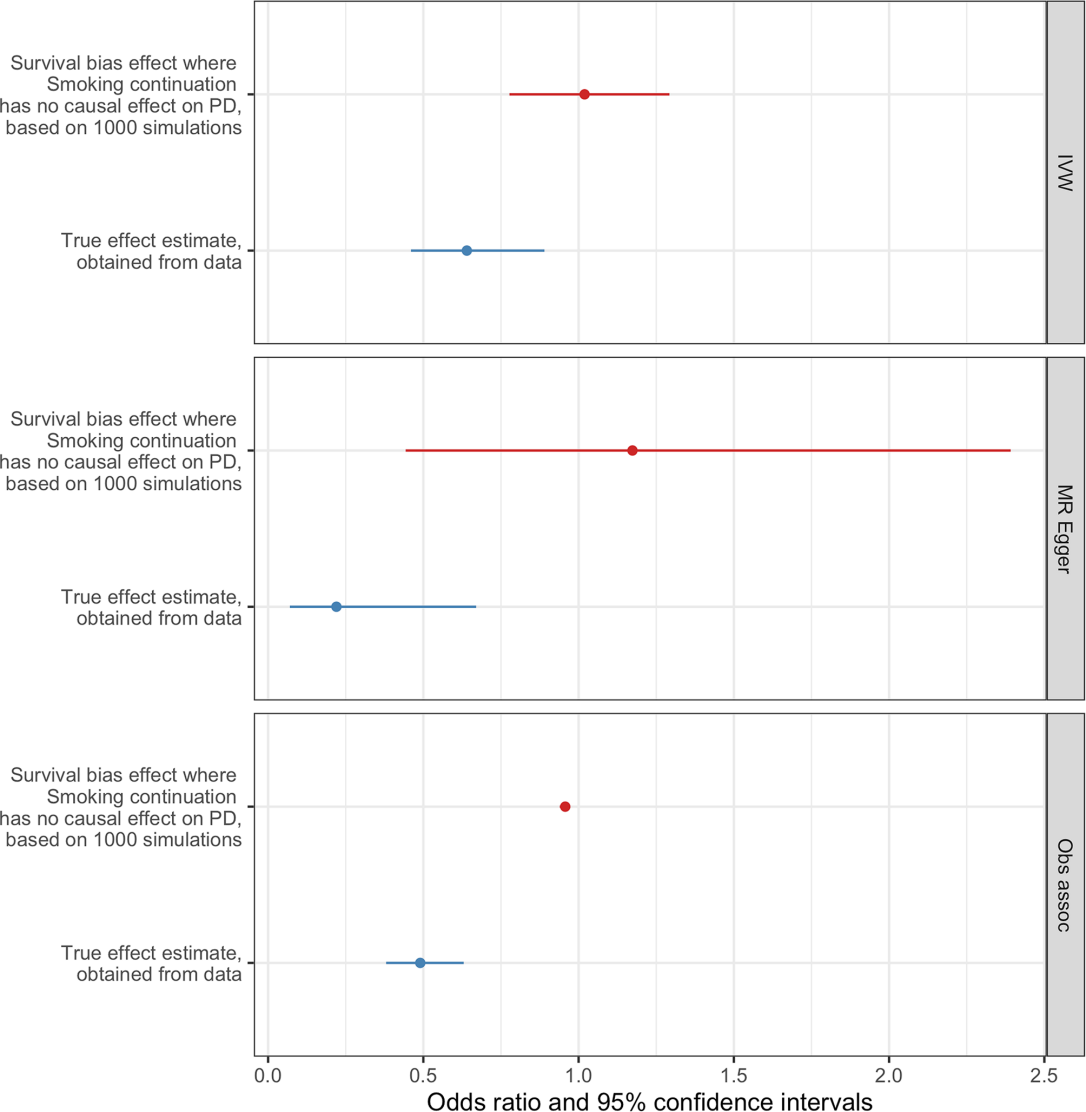
Frailty analysis results for *smoking continuation*. Comparison between the effect estimates obtained from simulations in which the effect *smoking continuation* has over PD risk are explained by the presence of survival bias (red) and the resulting effect estimates between *smoking continuation* and PD risk obtained from our MR analysis (blue), estimated for the IVW, MR-Egger and observational approaches.

Neurodegenerative disorders that are strongly associated with age, such as PD, may be subject to bias if selective mortality occurs. For example, if individuals who consume alcohol or continue smoking die prematurely before having a PD diagnosis, then those individuals who don’t consume alcohol or quit smoking might be more represented among PD patients. Thus, survival bias might explain the association between either alcohol consumption or being a current smoker and PD risk. In order to test if survival bias was sufficient to explain the MR results estimated from this study, we followed the methodology used by Noyce^35^, which consisted of a series of simulations that were performed to obtain the effect sizes that are expected from the frailty effects alone. If the effect sizes obtained from the frailty analysis were to be similar to the effect sizes obtained from the Mendelian Randomization analysis, then it would be suggested that the survival bias is large enough to explain the results. In the case of *drinks per week*, the simulations showed that there is a negligible influence of frailty effects biasing the MR results as the effect induced solely by frailty in the IVW analysis was an OR of 0.994 (95% CI 0.909–1.087) compared to the MR result OR of 0.78 (95% CI 0.69–0.88) (Fig. 5). On the other hand, for *smoking continuation* frailty effects do not exert a bias over the MR results as the effect of frailty alone in the IVW approach was 1.02 OR (95% CI 0.77–1.29) in contrast to the result from the MR analysis, which had a 0.64 OR (95% CI 0.46–0.89) (Fig. 6). Finally, we performed a one-sample test to determine if the difference between the IVW frailty analysis results and the estimated IVW MR values was significant. A p-value < 2.2e−16 was obtained for both *drinks per week* and *smoking continuation* traits, indicating that the difference between the frailty analysis results and our MR analyses are significantly different.

**Figure 7:**
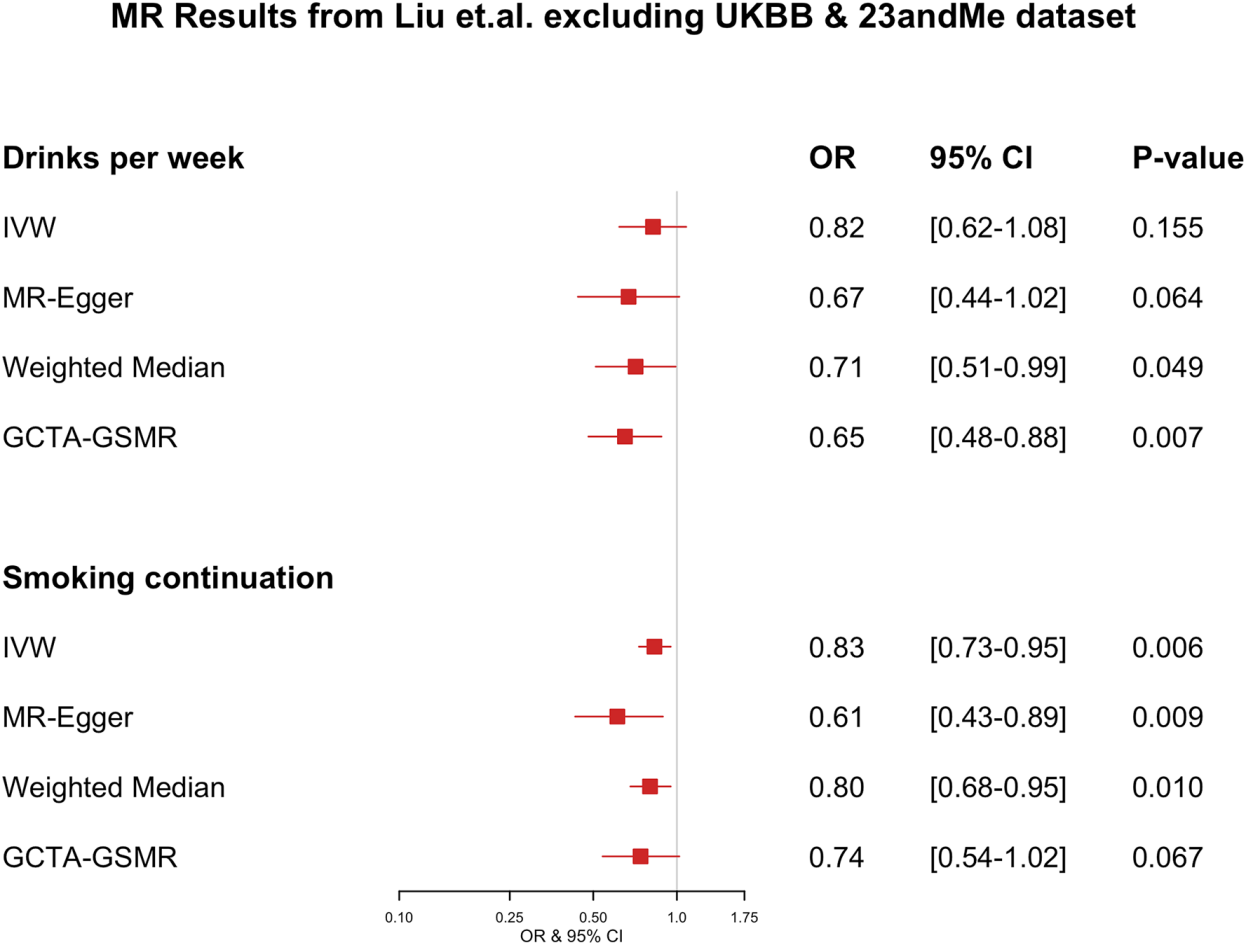
Forest plot showing OR and 95% confidence intervals of MR effect estimates calculated from the Liu et al*.* dataset that excluded UKBB and 23andMe. OR values for *smoking continuation* are expressed in *per doubling of odds* units.

Both the exposure and PD datasets include participant data from the UK Biobank (UKBB) and 23andMe cohorts. To test whether the two-sample MR findings might be biased due to the sample overlapping^36^, we derived genetic associations with the exposures of interest using a non-overlapping data subset^37^. We used a publicly available dataset by Liu et al*.*^28^*,* which excludes data from both UKBB and 23andMe. SNP-exposure estimates (for instruments used in our main analysis) from the GWAS conducted on these subsets were used to re-evaluate our MR associations. For *drinks per week*, the OR from the IVW estimate was 0.82 (95% CI 0.62–1.08; p = 0.155), and no evidence for horizontal pleiotropy (MR-Egger estimate of 0.67 (95% CI 0.44–1.02; p = 0.064) and an intercept value of 0.005 (p = 0.224)), and a GCTA-GSMR estimate of 0.65 (95% CI 0.48–0.88; p = 0.007) (see Fig. 7). For *smoking continuation*, the IVW estimate was 0.83 (95% CI 0.73–0.95; p = 0.006), with a MR-Egger estimate of 0.61 (95% CI 0.43–0.89; p = 0.009) with an intercept of 0.024 (p = 0.084), indicating no presence of horizontal pleiotropy, and the GCTA-GSMR estimate of 0.74 (95% CI 0.54–1.02; p = 0.067) (see Fig. 7). *Smoking initiation*, *smoking heaviness,* and *age at smoking initiation* showed similar associations as those reported in the main analysis but with wider confidence intervals. The complete findings from the analysis using the dataset excluding the 23andMe and UKBB samples can be found in Supplementary Fig. S4.

## Discussion

In the present study, we provide genetic evidence to support a protective role of moderate alcohol intake against PD risk (OR 0.79, 95% CI 0.65–0.96; p = 0.021). Moreover, we found that being *a current smoker* vs. *a former smoker* was causally associated with reduced PD risk (OR 0.64 95% CI 0.46–0.89; p = 0.008), whereas no causal association could be established for *smoking initiation*, *smoking heaviness,* and the *age at smoking initiation*.

Interestingly, in the case of alcohol intake, both the effect estimate and direction of causal association were consistent (OR 0.84, 95% CI 0.63–1.11; p = 0.216) even after removing the rs1229984 variant, which had the strongest association with *drinks per week* and corresponded to the *alcohol dehydrogenase 1B* locus*,* in which the reduction in precision that is shown by wider confidence intervals and a non-significant p-value is expected as the variant rs1229984 has the strongest association with the drinks per week phenotype, but the value of the causal estimate (0.84) remained in the same direction and is in fact very close to the original estimate (0.79) and therefore we can conclude that the effect that alcohol drinking has over PD risk is not solely driven by the variant rs1229984 and thus horizontal pleiotropy is not underlying the association*.* Nonetheless, the individual estimate for the effect of the rs1229984 variant over PD remained similar to the original result but with lower precision, indicated by broader confidence intervals (OR 0.75 95% CI 0.59–0.96; p = 0.025), which positions the rs1229984 SNP as a suitable candidate for investigating the mechanism behind the protective effects of alcohol intake on PD risk. The ADH1B protein metabolizes ethanol into acetaldehyde during alcohol metabolism, and the risk allele rs1229984 (A) produces a substitution of Arginine for a Histidine that changes affinity for the NAD+ coenzyme and increases ethanol oxidation by a 70–80-fold^38^. Individuals that carry the rs1229984 (A) allele, therefore, present a condition known as “alcohol flush reaction”, characterized by a visible flush on their face and body, and in some cases, symptoms such as nausea, headache, and general discomfort. Hence, carriers of the A allele tend to limit their alcohol intake. In line with our findings, a recent study by García-Martín et al*.*^39^ reported an inverse association between the reduced alcohol intake phenotype of the rs1229984 (A) allele and increased PD risk. Considering that this variant is both implicated in alcohol metabolism and strongly associated with alcohol intake, it is a promising candidate for investigating the mechanism by which alcohol drinking influences PD risk. Another strongly associated SNP with *drinks per week* was rs29001570 (see Supplementary Fig. S1), located in the *Alcohol Dehydrogenase 5 locus* (*ADH5*), which has also been previously associated with alcohol intake^40^. *ADH5* is known to be involved in formaldehyde metabolism but has a low affinity for alcohol, so the mechanism by which it associates with alcohol intake is not well understood.

The average level for *drinks per week* phenotype across Liu et al.^28^ meta-analysis is 7.6 drinks per week, which would broadly refer to a moderate drinking pattern according to the Dietary Guidelines for Americans 2015–2020^41^, which defines moderate alcohol consumption as up to 1 drink per day (7 drinks per week). It has also been shown that moderate alcohol consumers are at reduced risk of all-cause mortality [HR 0.78, 95% CI 0.74 to 0.82]^42^. This indicates that there is a low chance that survival bias may be affecting our *drinks per week* results, an idea that is further supported by our frailty analysis results.

We found that variants associated with being *a current smoker* in the *smoking continuation* GWAS were suggested to be causally associated with reduced PD risk. Although the IVW result showed evidence for an association with OR 0.64 (95% CI 0.46–0.89; p = 0.008) per doubling of odds on PD, the MR-Egger estimate suggested the presence of unbalanced horizontal pleiotropy, suggesting multiple potential (independent) biological modes of action linking the smoking variants onto PD risk, although the point estimate remain widely consistent with the IVW findings. Pleiotropic effects might be exerted in part by the lower genetic correlation between *drinks per week* and *smoking continuation* phenotypes reported by Liu et al.^28^ (r_g_ = 0.11, p = 6.45 × 10^–5^). However, it is unlikely that this correlation is solely responsible for the effect *smoking continuation* has on PD risk. Importantly, given that the discovery GWAS ignored time to cessation in the *former smokers* group, the phenotype can be conceivably more heterogeneous, making extrapolation of causality from these genetic-associations seemingly complicated. As the present study might lack sufficient power to characterize predictive instruments for *smoking continuation* (see Table 1), future studies leveraging even larger sample sizes for both *smoking continuation* and PD will be needed to validate these findings.

**Table 1:** The number of variants, R^2^, F-statistic and statistical power for each of the five phenotypes under investigation.

Exposure	Total SNPs	Sample size	R^2^	F-statistic	Power (%)
Age at smoking initiation	7	341,427	0.00102	49.74375	33
Smoking initiation	87	1,232,091	0.00441	62.74564	89
Drinks per week	33	941,280	0.00513	147.00378	93
Smoking continuation	8	547,219	0.00105	72.13881	34
Smoking heaviness	25	337,334	0.00983	113.90956	100

Power was calculated to detect a difference in PD risk with an OR of 0.8 in the IPDGC cases and controls with an alpha of 5%.

Notably, most smokers have attempted to quit smoking at least once in their lifetime^43^, and tobacco dependence is associated with longer smoking duration and failure of quitting attempts^44^. Therefore, *being a current smoker* could simply be interpreted as individuals being less likely to quit because they have a stronger genetic predisposition to smoking, whereas *former smokers* have a comparatively weaker predisposition and can quit successfully. Thus, our results support the hypothesis of smoking being a protective factor against PD.

It is important to note that the exclusion of never smokers in the *smoking continuation* phenotype (current vs. former smokers) influences the results’ interpretation. Our *smoking continuation* MR result suggests that prolonged smoking confers the protective effect against PD risk compared to shorter-term smoking. In contrast, the *smoking initiation* (ever vs. never smoker) result did not reach statistical significance. This might be because the “ever smoker” group contains both “current” and “former” smokers. The “former” smokers might be masking the protective effect seen in “current” smokers, therefore yielding a non-significant effect when the “ever smoker” group is compared against the “never smoker” group. Unfortunately, the Liu et al.^28^ study did not compare ‘never and former smokers’ vs. ‘current smokers’. We hypothesize that, under that scenario, smoking’s protective effect would be similar to that seen for *smoking continuation*. Future studies could investigate this question.

Weak instrument bias can arise when there is overlap between the two datasets used in the two-sample MR approach. The degree of overlap and its effects cannot be calculated, but it can potentially bias the resulting estimate towards the observational association^37^. To overcome the potential *weak instrument bias* due to overlapping samples in the exposure and outcome datasets (i.e., both include participants from the UKBB and 23andMe), we repeated the MR analysis using a Liu et al*.* data subset that excluded both UKBB and 23andMe. The results for both *drinks per week* and *smoking continuation* remained significant for most of the methods evaluated. For *drinks per week*, the OR from the IVW estimate was 0.82 (95% CI 0.62–1.08; p = 0.155), and for *smoking continuation*, IVW estimate was 0.83 (95% CI 0.73–0.95; p = 0.006). As we can conclude by comparing Figs. 2 and 7, the estimates across the four methods evaluated (IVW, MR-Egger, Weighted Median, GTCA-GSMR) for both *drinks per week* and *smoking continuation* were slightly shifted towards the null, and the 95% confidence intervals were widened. This observation was expected because removing the UKBB and 23andMe cohorts decreased the sample size from 1.2 million to ~ 250,000 participants. Such a reduction in sample size results in lower precision of the estimate shown by the widening of confidence intervals and the reduction of p-value significance. However, despite the considerable reduction in sample size, the resultant point estimates were similar to those reported in our main analysis. These findings provide additional assurance that our initial MR results were unlikely driven to be by inadequate sample overlap management.

Interpreting univariate MR results can be challenging because SNP instruments associated with the exposure of interest can exert pleiotropic effects on correlated risk factors. However, discarding SNPs based on an observed association with correlated traits can greatly diminish statistical power to detect an association. By conditioning out the association of each instrument on pleiotropic risk factors alongside the exposure of interest in our adjusted model, we evaluated the direct effect that our exposure of interest has on PD risk. While this approach is conceptually useful to tackle these issues, there are several potential concerns. Firstly, while results from the multivariable MR analysis conceptually help obtain a marginal estimate of the effect size between an exposure of interest and the outcome by conditioning competing risk factors, the model can potentially over-correct true associations as the underlying model does not distinguish between horizontal (independent of causality) and vertical (in line with causality) pleiotropy. For instance, the genetic associations with alcohol and smoking can be heavily confounded by (genetic effects on) socio-economic status^28^. In practice, the single SNP result for alcohol is likely a more reliable predictor for habitual alcohol intake given its well-understood role in regulating alcohol metabolism. However, unlike most variants, it is not associated with proxies of obesity, smoking, or educational attainment. Our results suggest that the protective relationship of alcohol intake (*drinks per week*) on PD remains consistent even after controlling for the (potential) pleiotropic effects of these SNPs on smoking behavior, obesity, and educational attainment. Thus, the causal association between alcohol intake and PD is unlikely to be the product of confounding via smoking behavior.

The frailty analysis we performed aimed to test if the results obtained from the MR analysis in this study could be explained by the effects of frailty alone, in which a survival bias due to selective mortality was responsible for the causal estimate between either alcohol consumption or *smoking continuation* and PD risk. We found that, in the case of *drinks per week*, survival bias introduced by the frailty effect is not sufficient enough to explain the magnitude of the association between *drinks per week* and PD risk shown by the MR results. Similarly, for smoking continuation, there was no evidence that the frailty effects were solely responsible for the estimate obtained from the MR analysis. Therefore, our frailty analysis results rule out that survival bias can explain the causal association between that either *alcohol intake* or *smoking continuation* and PD risk we found through the Mendelian randomization analysis.

A handful of recent studies have attempted to examine the causal relationship between smoking and alcohol intake on PD risk using Mendelian randomization. Nalls et al.^26^ reported little evidence for a causal relationship between PD and current smoking status (OR 0.545; 95% CI 0.230–1.291; p-value = 0.1681). Noyce et al*.*^27^ reported an inverse causal association between both current smoking status and increased alcohol intake and PD risk, but those associations were not explored in detail, and sensitivity analyses results were not explored or discussed in depth. Even though the Noyce et al*.* results and ours are similar, an important difference is the source of both the exposure (UKBB vs. Liu et al. 2019) and outcome (Chang et al*.* 2017 vs. Nalls et al*.* 2019) GWAS summary statistics datasets. The analyses presented here used datasets that comprised substantially larger and better-powered samples than those previously used. Also, we performed a series of sensitivity analyses to minimize the possibility of bias due to pleiotropy.

One limitation of the current study is that we could not perform subgroup analyses, for instance, to determine whether the protective effect of alcohol intake on PD risk is the same in both men and women. That is an important question as high-volume, and high-frequency drinking are more prevalent in men than women^45^. At the same time, we could not consider the survival effects of alcohol intake and smoking behavior. Heavy smoking increases mortality, so heavy smokers may be underrepresented among PD patients^46^. Finally, given that the PD GWAS consisted of a case–control design, the effects of smoking and drinking behavior can only be interpreted in the context of risk of developing PD, but are not informative of the effect that these behaviors might exert on disease progression of individuals with a current PD diagnosis.

Finally, it is crucial to be aware that smoking and drinking pose serious health risks to individuals. Evaluation and translation of these epidemiological clues for therapeutics have been difficult and thus far unsuccessful. However, a better understanding of the underlying biology and their relationship with PD risk could ultimately help delineate novel targets for prevention or treatment without the adverse health effects of smoking and drinking.

## Methods

### Data sources

We obtained GWAS summary statistics from the GWAS & Sequencing Consortium of Alcohol and Nicotine use (GSCAN) consortium (Liu et al.^28^ www.conservancy.umn.edu/handle/11299/201564), in which 556 genetic variants in 406 loci were associated with the following phenotypes: *smoking initiation (ever being a regular smoker), age at smoking initiation, smoking heaviness (cigarettes per day), smoking continuation (current vs. former smoker)*, and the number of *drinks per week,* in up to 1.2 million participants (Phenotype definitions can be found in the Supplementary Note). The beta coefficients, standard errors, and p-values were extracted from all five datasets. To address the potential instrumental bias issue, we obtained from the GSCAN consortium an additional summary statistics dataset, which excluded both UKBB and 23anMe and consisted of ~ 250,000 participants. The sample sizes for each GWAS study are listed in Table 1.

We also obtained summary statistics from the most recent PD GWAS from the International Parkinson’s Disease Genomics Consortium^26^ (IPDGC; www.pdgenetics.org/) and 23andMe Inc., through direct request. This dataset consisted of 7.8 million analyzed genetic variants in 37,688 cases, 18,618 UKBB proxy cases (first-degree relatives of PD patients), and 1.4 million controls.

### Statistical analyses

All datasets were “clumped” using PLINK (http://zzz.bwh.harvard.edu/plink/clump.shtml). Variants selected as index SNPs were those with the smallest p-values < 5e−8, while clumped SNPs were those in high linkage disequilibrium with the index SNP (R^2^ threshold of 0.001) or within a distance of 10,000 kb based on the 1000 Genomes project reference dataset. From the 406 variants identified by Liu et al.^28^ (10 associated with the *age at smoking initiation*, 55 with *smoking heaviness*, 378 with *smoking initiation*, 24 with *smoking continuation*, and 99 with *drinks per week*), we identified 7 associated variants with the *age at smoking initiation*, 29 with *smoking heaviness*, 96 with *smoking initiation*, 8 with *smoking continuation* and 42 with *drinks per week,* after clumping.

To evaluate whether the SNP instruments selected for each exposure were strongly associated with the exposure, the proportion of phenotypic variance for each trait explained by the SNPs (R^2^) and *F* statistics were calculated for the five traits (see Table 1). R^2^ for each trait was calculated based on the formula

$$\mathop \sum \limits_{i}^{{}} \frac{{2m_{i} \left( {1 - m_{i} } \right)\beta _{i}^{2} }}{{Var\left( Y \right)}},$$

where *m*_*i*_ is the minor allele frequency and β_i_ the magnitude of the association between the i-th SNP on trait Y^47^. The *F statistic* was calculated as previously described by Noyce et al*.*^35^:

$$F = \frac{{R^{2} \left( {n - 1 - k} \right)}}{{\left( {1 - R^{2} } \right)~ \times ~k}},$$

where *n* is the sample size and *k* the number of SNPs.

The beta coefficients, standard errors, and p-values were extracted from the PD dataset for those index SNPs previously identified in each study. We then inspected how many index SNPs were shared between PD and each of the five traits. In the case of the *age at smoking initiation* and *smoking continuation*, all index SNPs were also found in the PD dataset (n = 7 and 8, respectively). Moreover, 25 SNPs were found for the *smoking heaviness*, 87 SNPs for *smoking initiation*, and 33 SNPs for *drinks per week*. No suitable LD-proxies were found in the PD dataset for any of the missing variants. For the bidirectional-MR, 88 index SNPs were identified from the PD GWAS dataset and further extracted from the *smoking continuation*, *smoking initiation,* and *drinks per week* GWAS datasets.

Power calculations were conducted using the method by Brion et al*.*^48^ (shiny.cnsgenomics.com/mRnd/) and the R^2^ previously calculated, in which the power to detect an effect of OR 0.8 on PD risk in the iPDGC dataset with an alpha of 5% was evaluated for each trait (see Table 1). *Smoking heaviness* showed the greatest percentage of power with 100%, followed by *drinks per week* with 93% and *smoking initiation* with 89%. The traits with the lowest power values were *smoking continuation* and *age at smoking initiation* with 34% and 33%, respectively.

To evaluate potential instrumental bias, we extracted the beta coefficients, standard errors, and p-values from the excluding UKBB and 23andMe datasets for those index SNPs for each trait previously identified in the original Liu et al*.*^28^ dataset. Finally, we repeated the MR analyses with this new smoking and drinking dataset and the same PD dataset as the previous analysis.

### Effect allele harmonization

Harmonization across both the GSCAN and PD datasets was carried out as described by Hartwig et al.^36^ before the MR analyses. First, all beta coefficients of the smoking phenotypes datasets were ensured to be positive (to facilitate interpretation of plots and as a requirement for further MR analysis). Negative beta coefficients were ‘flipped,’ e.g., they were multiplied by − 1, the effect allele became the alternative allele, and the effect allele frequency was subtracted from 1. Then, we compared the effect allele coding in the smoking dataset and the Parkinson’s disease dataset and ensured that both had the same allele coding. Variants in the PD dataset with different effect alleles in the smoking dataset were ‘flipped’ as previously described.

### Mendelian randomization analyses

Before the MR analyses, we first harmonized both the GSCAN and PD datasets to correctly align the effect alleles. Four different MR methods were then selected to conduct the analysis: Inverse Variance Weighted (IVW)^49^, MR-Egger^50,51^, MR-PRESSO^52,53^ and GTCA-GSMR^25,53^, each with unique characteristics. A fundamental assumption required for MR inferences to be valid is that the instrument (SNP) and the outcome must be associated only through the exposure. If this assumption is violated, the presence of horizontal pleiotropy (an SNP associated with some other trait besides our exposure of interest) may bias MR estimates, potentially leading to erroneous conclusions^20,22,54^. In the case of the IVW model, it calculates the causal estimate by using a weighted linear regression that combines each instrumental variable’s wald ratios and weighting them by the inverse variance of the gene-outcome association while constraining the intercept to zero; therefore, it can only be interpreted under strong assumptions on the absence of pleiotropy^49^. The other three models exploit the statistical properties of MR estimates that are less vulnerable to these biases. For instance, MR-Egger provides a conservative estimate of the MR effect size in the presence of directional pleiotropy by fitting an intercept term to model the bias. If the intercept term is significantly different from zero, this is taken as evidence for horizontal pleiotropy^50,51^. MR-PRESSO identifies pleiotropic outliers that potentially contribute towards genetic heterogeneity under an IVW framework through non-parametric approaches^52^. In contrast, the GTCA-GSMR framework is a parametric-based method that derives the MR estimate using a generalized two-step least squares (2SLS) approach while accounting for pleiotropic outliers with the HEIDI-outlier technique^25^. Applying these models in parallel can help triangulate MR inferences through parametric and non-parametric approaches, making results more robust and less likely to be affected by weak violations of MR assumptions. We also generated funnel plots to inspect the presence of pleiotropic outliers manually, as asymmetry in the distribution of effect sizes indicates unbalanced horizontal pleiotropy. Cochran’s Q and I^2^ statistics were calculated to test for heterogeneity across estimates^50,51^.

To facilitate the interpretation of the effect estimates from binary variables, the odds ratios were multiplied by 0.693 to obtain the OR ‘per doubling of odds’, a concept introduced by Burgess et al.^55^ that refers to a doubling in the prevalence of the binary exposure.

### Multivariable MR analysis adjusting for competing substance use phenotypes

While our univariate MR analyses (i.e., fitting our exposure of interests one at a time) indicate whether genetic predisposition to these risk factors is associated with PD risk, it remains unclear whether other highly correlated environmental risk factors potentially mediate those relationships. To evaluate the direct effect between our exposures of interest and PD risk, we fitted another IVW regression model by regressing out the genetic association between our SNP instruments (for each trait respectively) and the following putative risk factors in our MR analyses: BMI, years of education, smoking status and cigarettes per day (for the DPW MR only); alcohol intake (for the smoking-related trait MR only). For BMI (kg/m^2^) and education attainment (in SD units), we derived the genetic effect size estimates from the UKBB cohort. The UKBB data were QC-ed as per previous work^47^: The GWAS for BMI (UKB data-field ID: 21001) included 437,458 individuals, while normalized educational attainment (UKB data-field ID: 6138) included 214,999 individuals. We used BOLT-LMM^56^ mixed model software adjusting for recruitment age, sex and the first ten ancestral principal components. For alcohol and smoking-related traits, we adopted the effect size estimates from the GSCAN summary statistics.

### Frailty analysis

We evaluated if survival bias could explain the MR results obtained from this study by following the methodology described by Noyce et al*.*^35^ in which the model assumptions are that either the *drinks per week* or *smoking continuation* are associated with mortality and PD is associated with age. In summary, the approach consisted in the following steps:

1. Simulating a large population (n = 6 million) where individuals had alleles at each evaluated SNPs (either 31 for *drinks per week* or 7 for *smoking continuation*).
2. Values for *drinks per week* or *smoking continuation* were simulated based on mean and sd values reported by Liu et al.^28^.
3. Age values were generated to match the age distributions reported in Nalls et al.^26^.
4. Influence of our traits of interest on survival was stimulated following an all-cause mortality hazard ratio of 1.12 for *heavy alcohol consumption*^42^ and 1.62 for *current smokers*^57^ used to generate a Gompertz–Makeham mortality curve. Alive-death status was then sampled from the probability of death obtained from the survival curve.
5. Simulation of PD status was based upon the age-related diagnosis reported from Driver et al*.*^58^.
6. A sample of 56,306 simulated cases with PD and 1.4 million controls following the age distributions reported by Nalls et al*.*^26^ was extracted. Finally, observational associations and MR analysis were performed over the simulated individuals. The known observational effect estimates were extracted from those reported by Zhang et al*.*^10^ for *alcohol consumption* and Gallo et al*.*^14^ for *smoking continuation*.
7. This process was repeated 1000 times to obtain the effect size distribution that can be attributable to frailty effects alone.

Code for the frailty analysis is accessible in the github repository.

## Supplementary Information

Supplementary Information.

## Data Availability

The data that support the findings of this study are available from the corresponding author upon reasonable request. To apply and access 23andMe summary statistics, please visit http://research.23andme.com/dataset-access/ for more information.

## Code availability

All code for this study is available at our GitHub repository: http://github.com/cdomingu/MRandPD.
