# Supplementary material for: Understanding the effect of smoking and drinking behavior on Parkinson's disease risk: a Mendelian randomization study
Source: Sci Rep. 2021 Jul 7;11:13980. doi: 10.1038/s41598-021-93105-y (PMC8263722; doi:10.1038/s41598-021-93105-y)
Supplement: Supplementary file 1 — Supplementary Information. [file 41598_2021_93105_MOESM1_ESM.pdf]

Genetic evidence for a protective effect of smoking and drinking behavior on Parkinson's disease risk: A Mendelian randomization study

Carmen Domínguez-Baleón[1], Jue-Sheng Ong[2], Clemens R. Scherzer[1,3,4,5], Miguel E. Rentería[1,2*] and Xianjun Dong[1,3,5,6*]

[1] Center for Advanced Parkinson Research and Precision Neurology Program, Brigham & Women's Hospital, Harvard Medical School, Boston, MA, USA.

[2] Department of Genetics & Computational Biology, QIMR Berghofer Medical Research Institute, Brisbane QLD Australia.

[3] Department of Neurology, Massachusetts General Hospital, Boston, MA, USA.

[4] Department of Neurology, Brigham and Women's Hospital, Boston, MA, USA.

[5] Genomics and Bioinformatics Hub, Brigham and Women's Hospital, Boston, MA, USA.

*These authors jointly supervised this work:

Correspondence: Dr. Xianjun Dong (xdong@rics.bwh.harvard.edu) & Dr. Miguel E. Rentería (miguel.renteria@qimrberghofer.edu.au).

SUPPLEMENTARY INFORMATION

Supplementary Note

Phenotype Definitions

Phenotypic terms for all the traits evaluated in this study were those used by Liu *et al*.[28], in which the authors defined each phenotype by the following definitions:

In the case of continuous traits, *age at smoking initiation* was defined by the age at which a participant started smoking with regularity, *smoking heaviness* as the average number of cigarettes smoked per day either as a former or current smoker, and *drinks per week* as the average number of drinks an individual reported drinking weekly regardless the alcohol type. *Smoking initiation* (ever vs. never smoker) was defined as a binary trait. Any participant that reported ever being a regular smoker (either current or former) was coded as a 'case', and any participant who reported never being a regular smoker was coded as a 'control'. This was measured by affirmative answers to any of the following questions: *Have you ever smoked over 100 cigarettes in your lifetime? Have you smoked daily for at least a month? Have you ever smoked regularly?*.

The *smoking continuation* trait was defined as a binary phenotype with current smokers coded as 'cases' and former smokers as 'controls'. Participants were assigned to either the "current" or "former" groups depending on their affirmative answers to both questions from the following combinations: *Do you currently smoke?* and *Have you ever smoked regularly?*, or *Do you smoke?* and H*ave you smoked over 100 cigarettes in your lifetime?*.

Supplementary Figure S1. Funnel plot for *drinks per week* effect estimates vs. standard errors. Overall symmetry of the plot indicates no unbalanced horizontal pleiotropy affecting the estimate. The highest point (the one with the smallest standard error), corresponds to variant rs1229984 located in the *ADH1B* locus, which presents the strongest association with *drinks per week*.

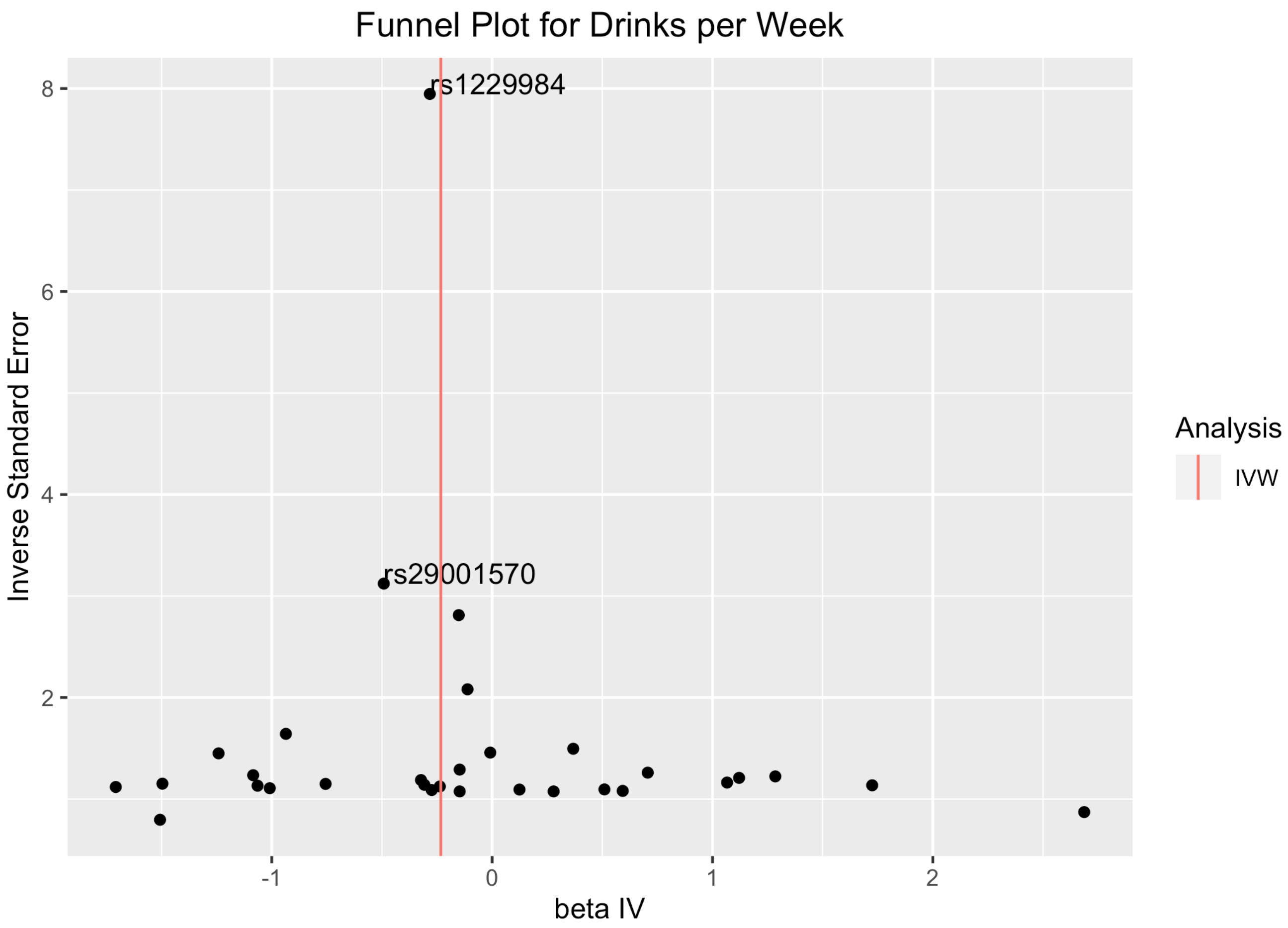

Supplementary Figure S2. Funnel plot for *smoking continuation* effect estimates vs. standard errors. As this phenotype is explained by only 7 index SNPs, no strong conclusion about potential unbalanced horizontal pleiotropy can be driven from the funnel plot.

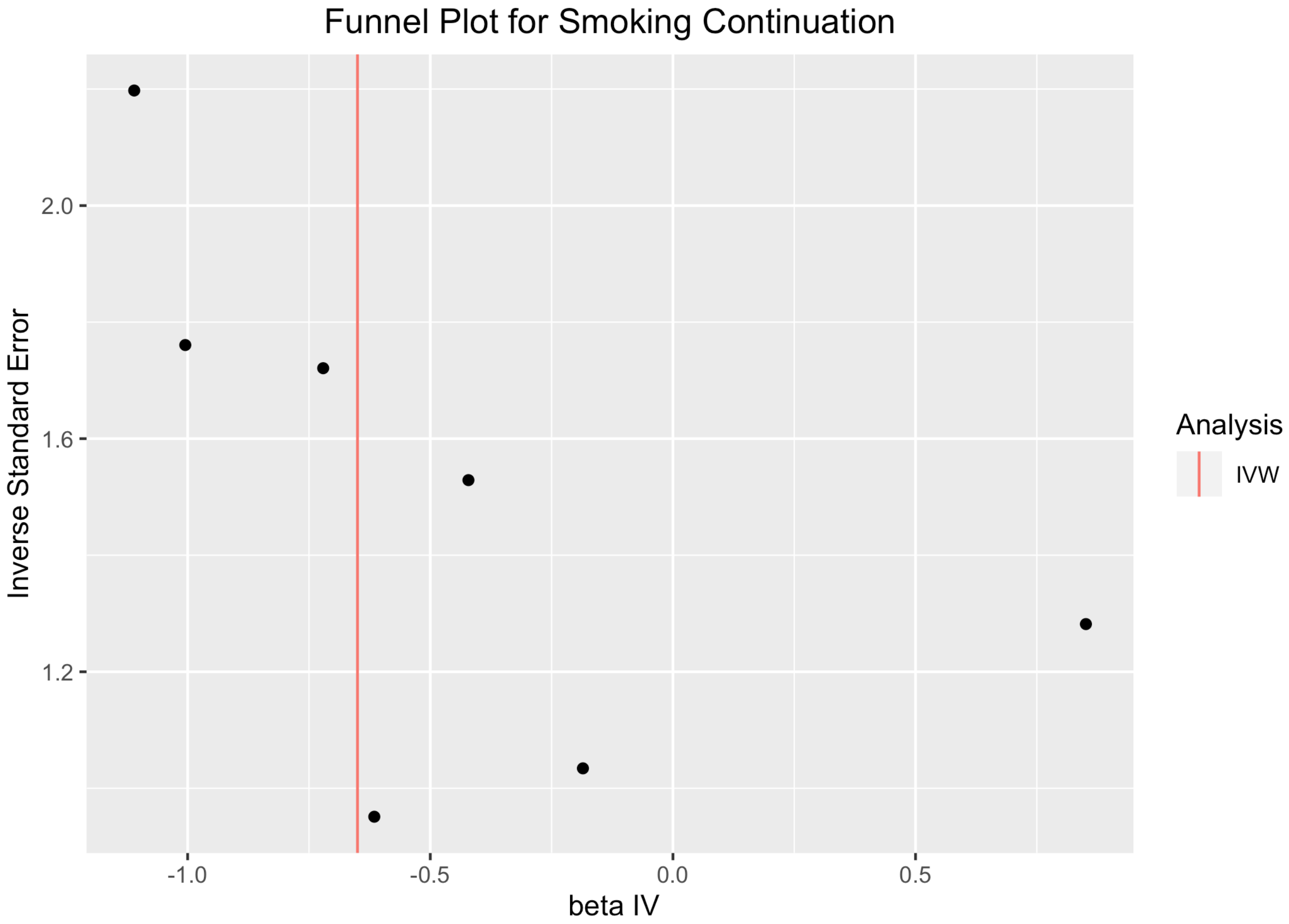

Supplementary Figure S3. Forest plot showing OR and 95% confidence intervals of MR effect estimates for the rest of smoking traits evaluated in this study. OR values for *smoking initiation* are expressed in *per doubling of odds* units.

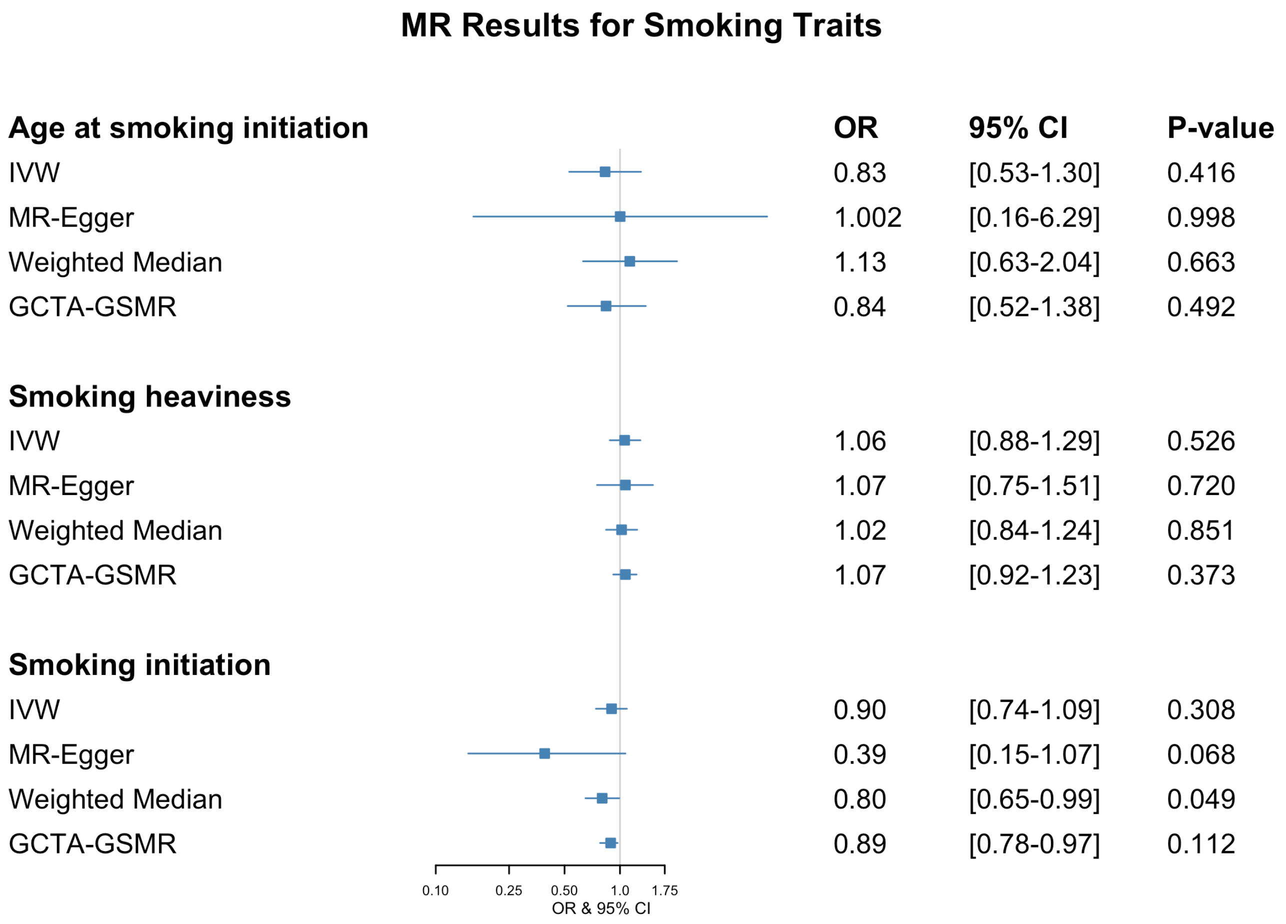

Supplementary Figure S4. Forest plot showing OR and 95% confidence intervals of MR effect estimates for the rest of smoking traits calculated from the Liu *et al.* dataset that excluded UKBB & 23andMe. OR values for *smoking initiation* are expressed in *per doubling of odds* units.

MR Smoking traits results from Liu et.al. excluding UKBB & 23andMe data

	OR	95% CI	P-value
**Age at smoking initiation**			
IVW	0.75	[0.44-1.28]	0.294
MR-Egger	0.61	[0.11-3.22]	0.560
Weighted Median	0.76	[0.38-1.52]	0.441
GCTA-GSMR	0.80	[0.42-1.54]	0.515
**Smoking heaviness**			
IVW	1.11	[0.92-1.33]	0.278
MR-Egger	1.05	[0.75-1.48]	0.763
Weighted Median	1.02	[0.84-1.24]	0.810
GCTA-GSMR	1.01	[0.85-1.19]	0.903
**Smoking initiation**			
IVW	0.95	[0.87-1.03]	0.228
MR-Egger	0.85	[0.62-1.17]	0.327
Weighted Median	0.91	[0.82-1.01]	0.070
GCTA-GSMR	0.87	[0.75-1.01]	0.073

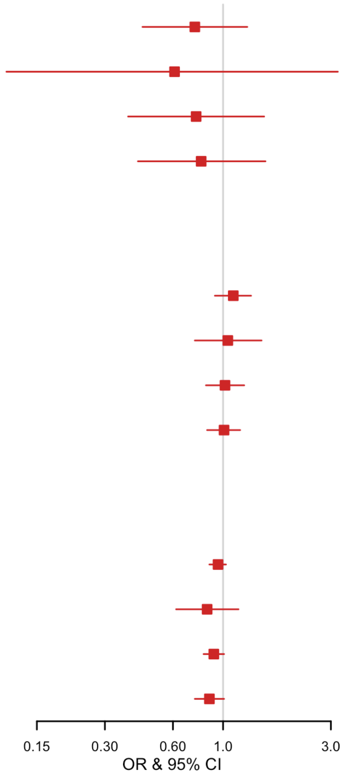

Figure S5. Scatter plot showing MR effect estimates of *smoking initiation* over PD. Each SNP-PD association is plotted against SNP-*smoking initiation* association and corresponding MR estimates for IVW, MR-Egger, Weighted-Median and GSMR are plotted.

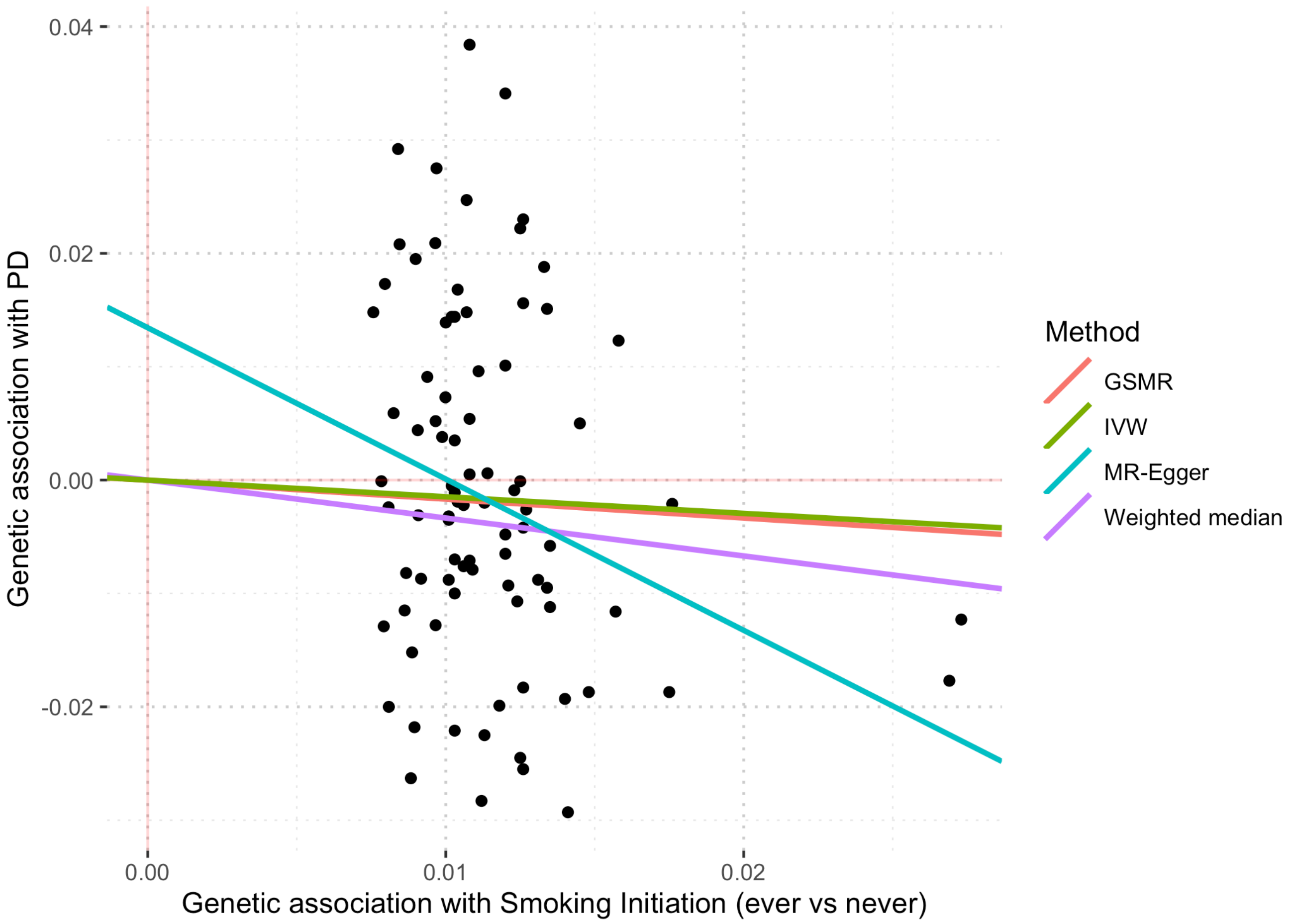

Figure S6. Scatter plot showing MR effect estimates of the *age at smoking initiation* over PD. Each SNP-PD association is plotted against the SNP-*age at smoking initiation* association and corresponding MR estimates for IVW, MR-Egger, Weighted-Median and GSMR are plotted.

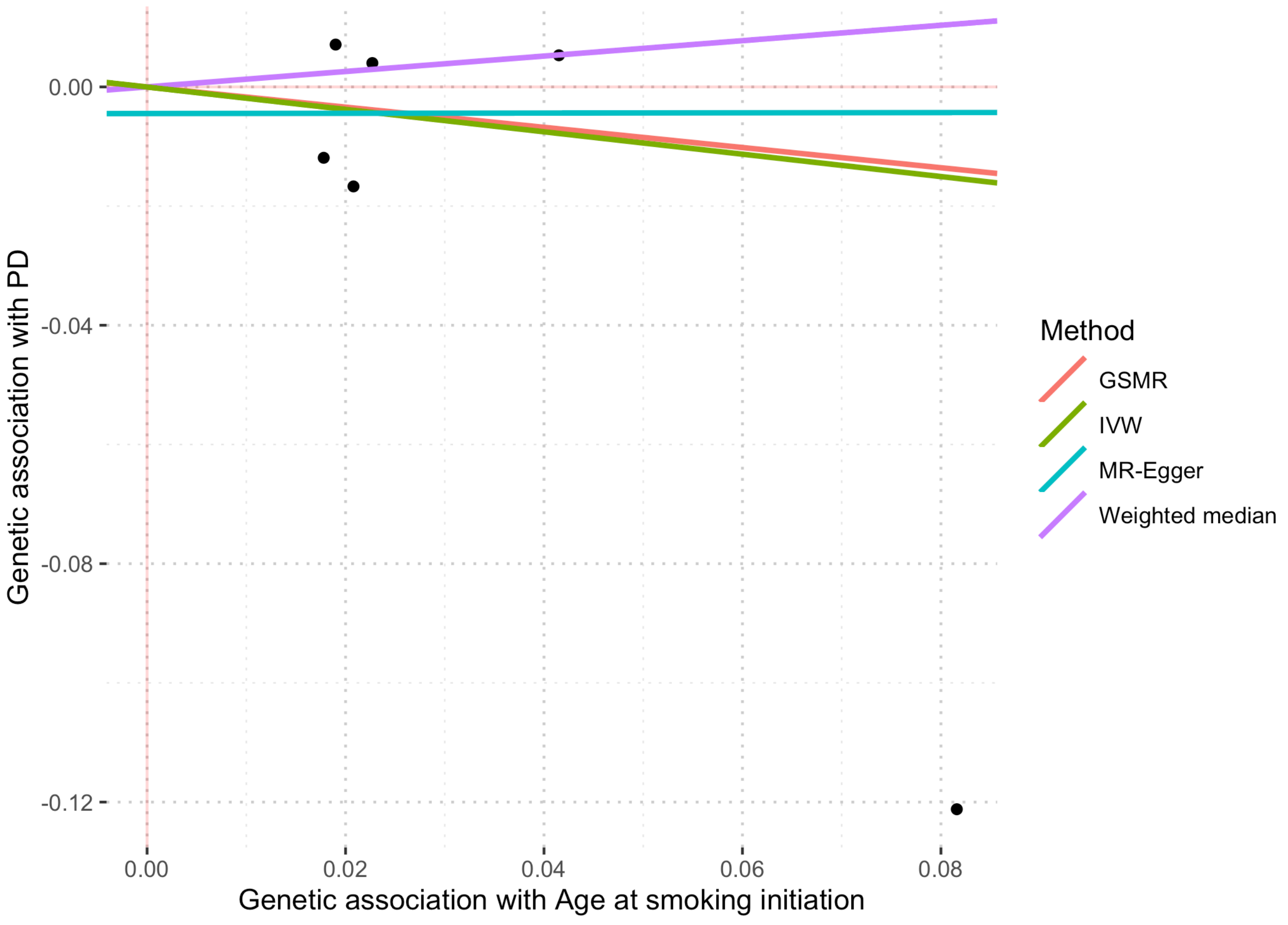

Figure S7. Scatter plot showing MR effect estimates of *smoking heaviness (cigarettes per day)* over PD. Each SNP-PD association is plotted against the SNP-*smoking heaviness* association and corresponding MR estimates for IVW, MR-Egger, Weighted-Median and GSMR are plotted.

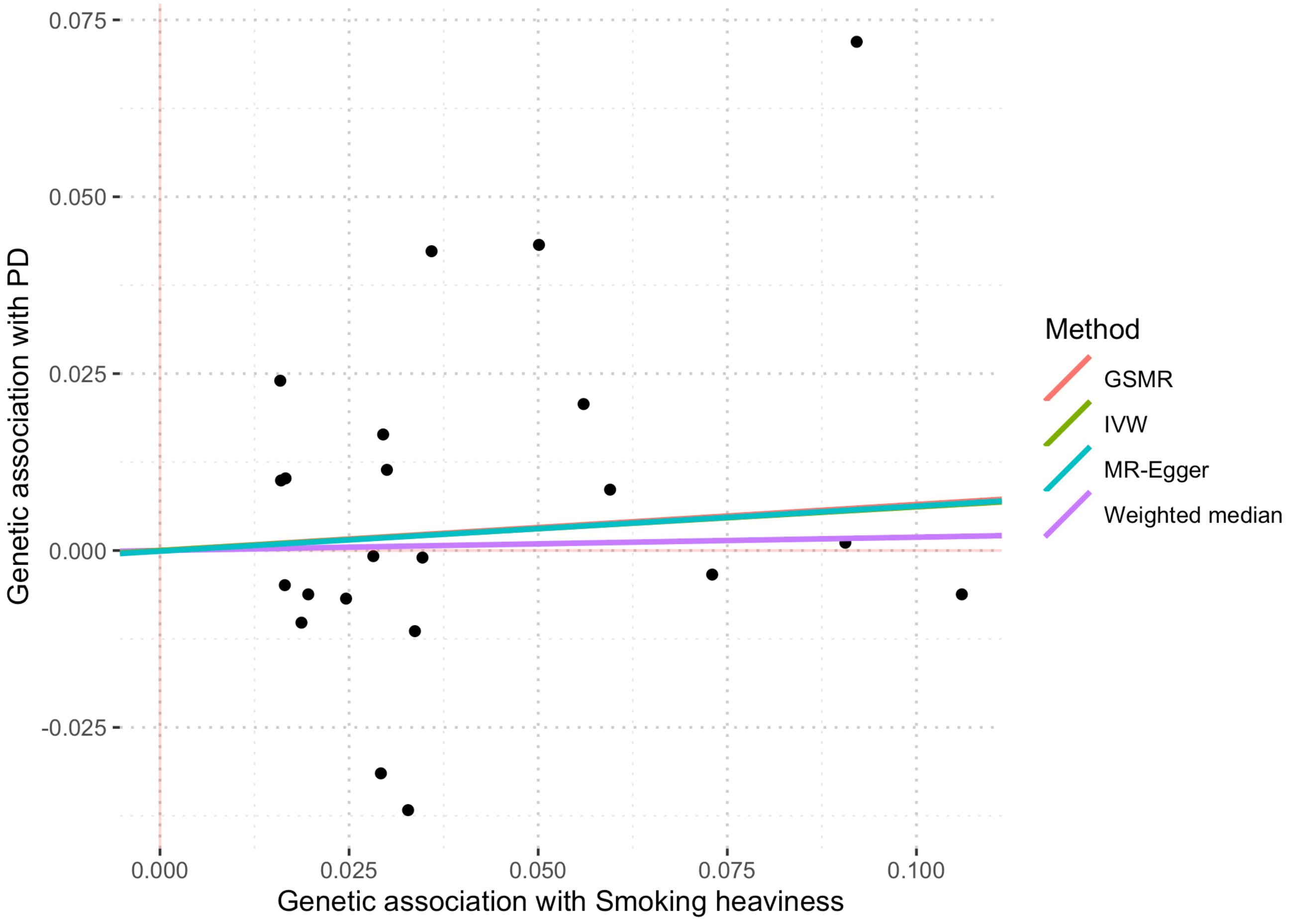

Supplementary Figure S8. Funnel plot for *smoking initiation* effect estimates vs. standard errors. Overall symmetry of the plot indicates no unbalanced horizontal pleiotropy affecting the estimate.

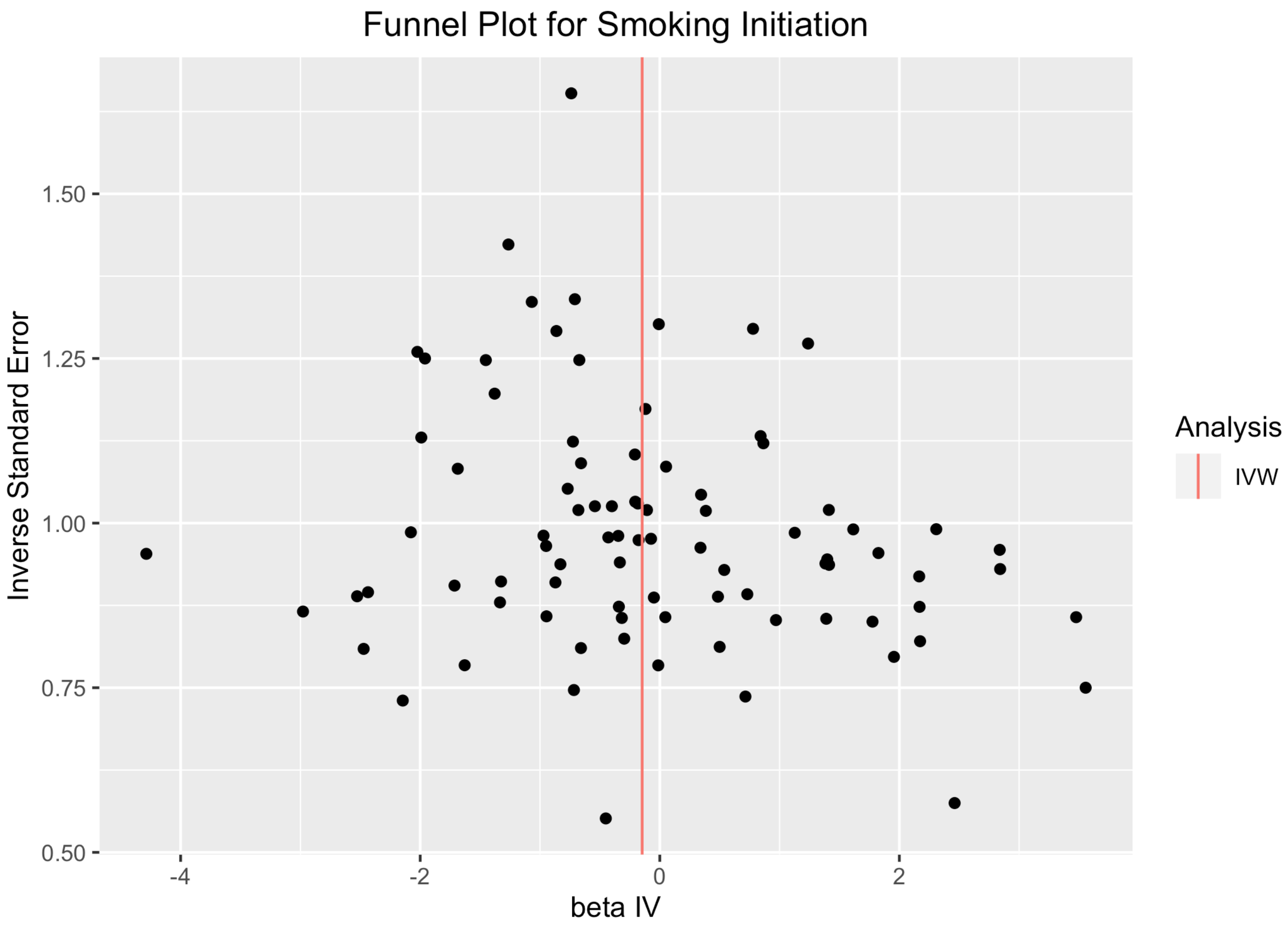

Supplementary Figure S9. Funnel plot for the *age at smoking initiation* effect estimates vs. standard errors. As this trait is explained by only 6 SNPs, no strong conclusion about potential unbalanced horizontal pleiotropy can be driven from the funnel plot.

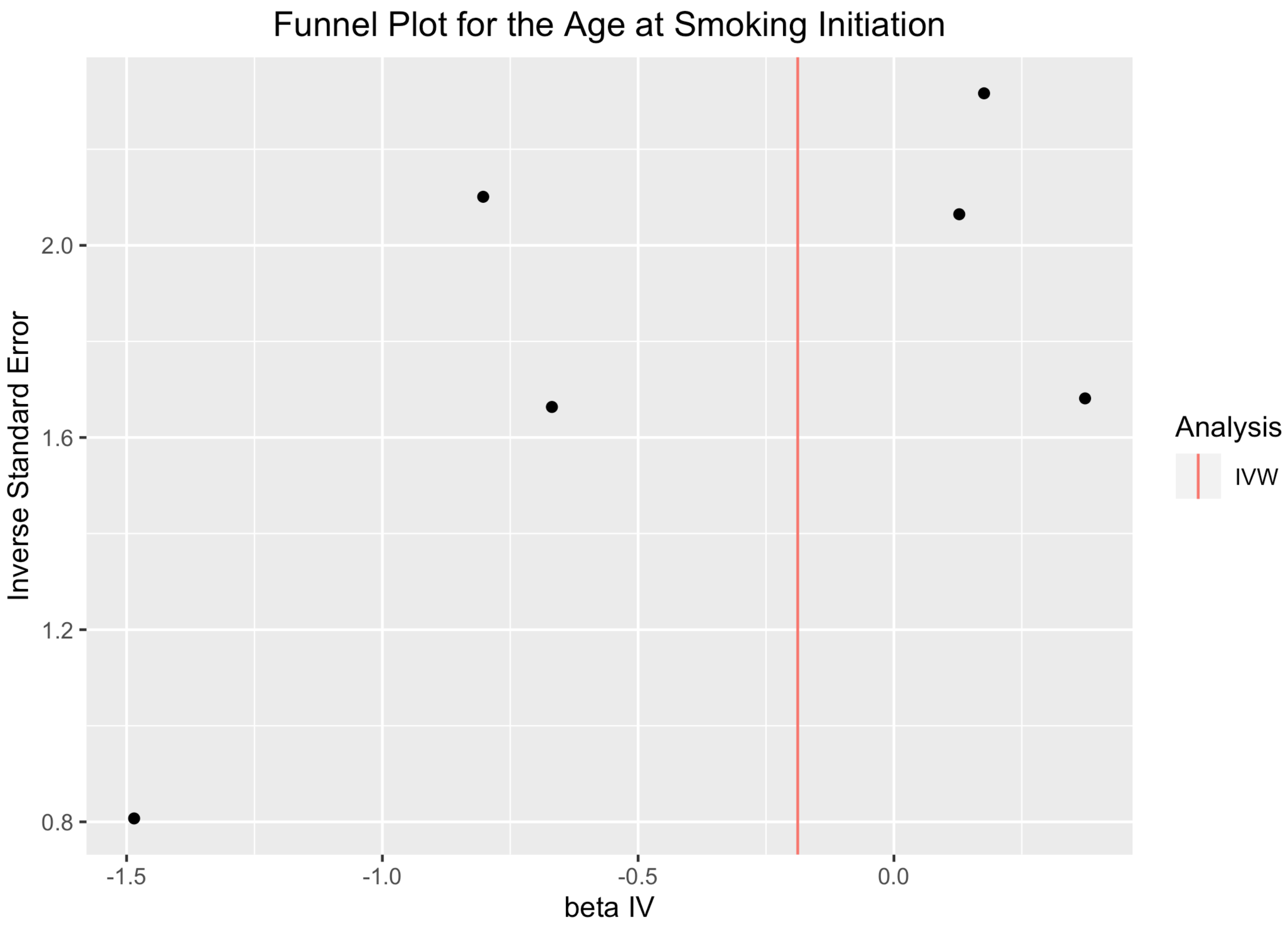

Supplementary Figure S10. Funnel plot for *heaviness of smoking (cigarettes per day)* effect estimates vs. standard errors. Symmetry around the point estimate indicates no unbalanced horizontal pleiotropy affecting the result.

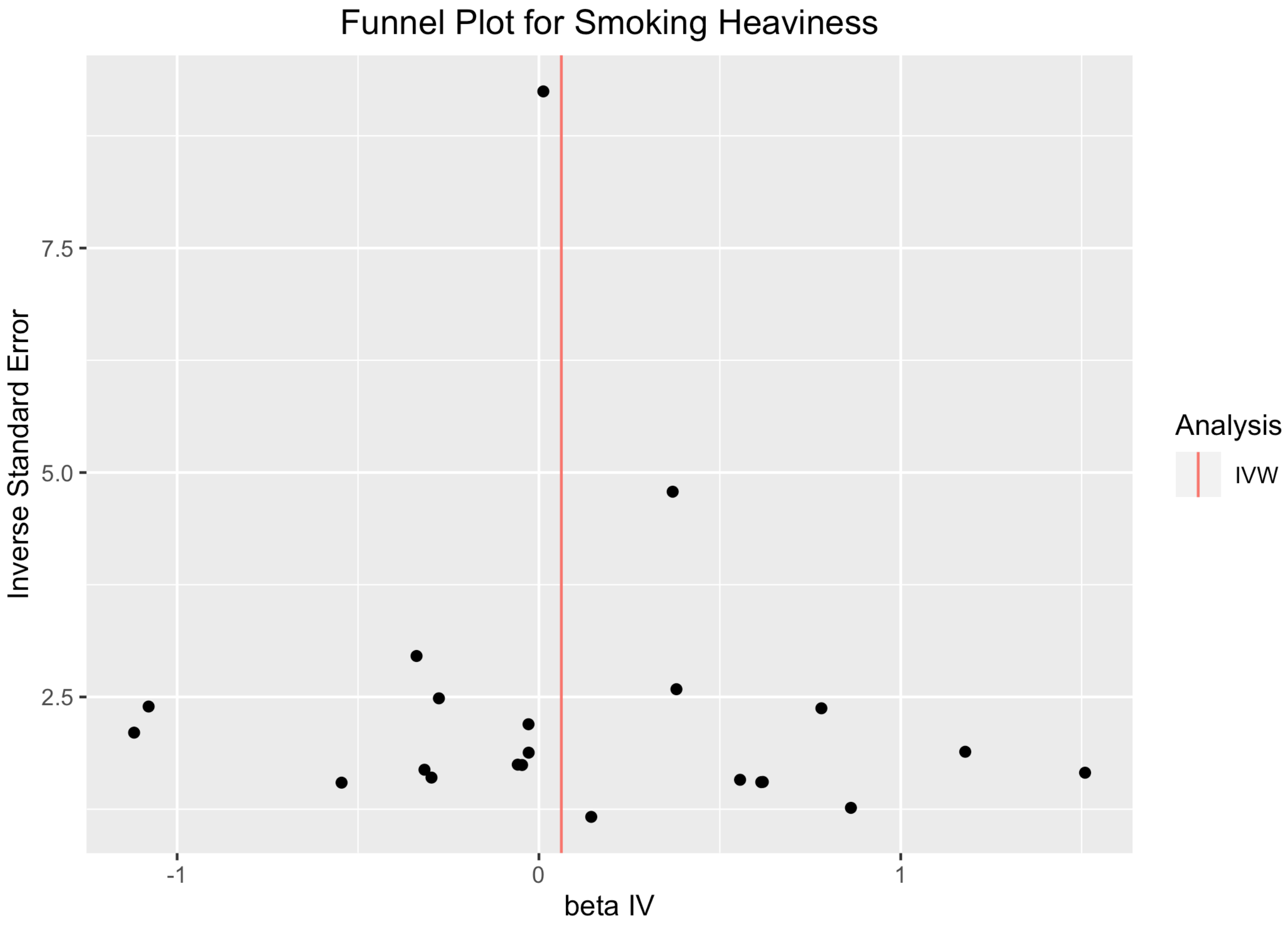

Supplementary Table S1. Variants associated with *Drinks per week* phenotype, their magnitude of effect on DPW and the strength of association with PD.

SNP	Chr	BP	EA	OA	EA freq	DPW beta	SE DPW beta	p-value DPW association	PD beta	SE PD beta	p-value PD association
rs10085696	7	69783020	A	G	0.799	0.016	0.00249	1.24E-10	0.0113	0.0127	0.3741
rs10173720	2	27855397	T	A	0.227	0.0131	0.00231	9.33E-09	-0.0099	0.0114	0.3827
rs1123285	14	57274519	C	G	0.661	0.0127	0.00208	1.36E-09	-0.0041	0.0107	0.7011
rs113909752	4	100281067	C	T	0.921	0.0385	0.00408	2.76E-21	-0.0043	0.0185	0.8158
rs11860773	16	73912503	T	C	0.824	0.0155	0.00251	8.35E-10	-0.0165	0.0137	0.23
rs1229984	4	100239319	C	T	0.953	0.209	0.00673	1.60E-203	-0.0591	0.0263	0.02449
rs1260326	2	27730940	C	T	0.595	0.0233	0.00196	3.33E-33	0.0236	0.0095	0.01293
rs13332432	16	85721809	G	C	0.296	0.0142	0.00219	5.94E-11	-0.0154	0.0115	0.1815
rs1387766	12	92081800	G	A	0.378	0.0108	0.00198	4.79E-08	0.0064	0.01	0.52
rs1826908	4	100301244	C	T	0.106	0.0239	0.00307	4.66E-15	-2e-04	0.0164	0.9917
rs2049045	11	27694241	G	C	0.811	0.0137	0.00251	3.97E-08	-0.0205	0.0119	0.0846
rs2299409	7	103812171	G	A	0.507	0.0104	0.00192	4.80E-08	0.0053	0.0095	0.5743
rs2303205	16	85722356	T	C	0.282	0.014	0.00229	7.80E-10	-0.0211	0.0176	0.231
rs28601761	8	126500031	G	C	0.405	0.0116	0.00201	7.60E-09	0.013	0.0096	0.1738
rs28680958	1	173848808	G	A	0.77	0.0136	0.00237	9.78E-09	0.0145	0.0117	0.2131
rs28712821	4	39413780	A	G	0.594	0.0284	0.00199	1.10E-46	-0.0043	0.0101	0.6678
rs28732378	3	85403892	A	G	0.271	0.0163	0.00217	2.24E-14	0.006	0.0109	0.5835
rs28929474	14	94844947	C	T	0.9846	0.0477	0.00719	2.39E-11	0.0613	0.039	0.1159
rs29001570	4	99994405	T	C	0.991	0.227	0.0134	9.71E-63	-0.1116	0.0727	0.1248
rs331939	4	143654889	G	A	0.661	0.0118	0.00202	4.50E-09	-0.0028	0.0105	0.7929
rs4233567	2	144272376	C	T	0.66	0.013	0.00208	3.83E-10	-0.004	0.0114	0.7216
rs4752999	11	47428565	C	T	0.679	0.0145	0.00207	2.03E-12	-0.018	0.01	0.07108

rs4788213	16	30034524	T	C	0.544	0.0109	0.00196	3.30E-08	0.0188	0.0096	0.05035
rs4916723	5	87854395	A	C	0.596	0.0115	0.00199	8.07E-09	0.0309	0.0132	0.01906
rs528301	2	45154908	A	G	0.605	0.0156	0.00195	1.25E-15	-0.0146	0.0095	0.1237
rs55872084	5	155902003	T	G	0.218	0.0129	0.00228	1.98E-08	0.0016	0.0118	0.8936
rs55932213	9	108755622	G	A	0.701	0.0129	0.0023	1.80E-08	0.0036	0.012	0.7619
rs58352691	4	42117070	T	G	0.0619	0.0218	0.00384	1.41E-08	-0.022	0.0197	0.2649
rs6106989	20	25027630	A	G	0.628	0.0113	0.00204	3.81E-08	-0.0193	0.0101	0.05547
rs6739804	2	63269604	T	C	0.34	0.0129	0.00208	4.72E-10	-0.0019	0.01	0.8522
rs6951574	7	153489744	C	T	0.459	0.0135	0.00205	4.44E-11	-0.0037	0.0124	0.7644
rs76217384	2	144200697	G	A	0.203	0.0129	0.0023	2.41E-08	-0.0019	0.012	0.8736

Supplementary Table S2. Variants associated with *smoking continuation (current vs former smoker)* their magnitude of effect and the strength of association between SmkC and PD.

SNP	Chr	BP	EA	OA	EA freq	SmkC beta	SE SmkC beta	p-value SmkC association	PD beta	SE PD beta	p-value PD association
rs10993863	9	136486815	G	A	0.602	0.0151	0.00291	3.67E-11	-0.0028	0.0146	0.8481
rs3025327	9	136467344	C	G	0.0935	0.0345	0.00405	1.96E-24	-0.0383	0.0157	0.01458
rs518425	15	78883813	A	G	0.693	0.0159	0.0027	7.57E-11	-0.0067	0.0104	0.5172
rs56113850	19	41353107	T	C	0.432	0.0206	0.00252	2.52E-26	-0.0207	0.0117	0.07728
rs6011779	20	61984317	C	T	0.214	0.0222	0.00314	2.04E-16	-0.016	0.0129	0.2142
rs60749569	8	42602668	A	T	0.9243	0.0234	0.00466	1.95E-08	-0.0144	0.0246	0.5589
rs7127006	11	16379226	A	G	0.278	0.0141	0.00272	2.24E-08	0.012	0.011	0.2789

Supplementary Table S3. Variants associated with *smoking initiation* (SmkInit), their magnitude of effect and their strength of association with PD.

SNP	Chr	BP	EA	OA	EA freq	SmkInit beta	SE SmkInit beta	p-value SmkInit association	PD beta	SE PD beta	p-value PD association
rs10001365	4	147797214	G	A	0.595	0.0106	0.0018	6.65E-12	-0.0022	0.0096	0.8212
rs1004787	2	45159091	A	G	0.581	0.0124	0.00178	5.27E-17	-0.0107	0.0096	0.2613
rs10159545	10	21766969	G	C	0.375	0.0126	0.00186	1.84E-12	-0.0183	0.0101	0.07128
rs10279261	7	133589846	G	A	0.381	0.0101	0.00187	5.00E-09	-0.0035	0.0103	0.7379
rs1029984	2	60526747	T	G	0.58	0.0103	0.0018	6.26E-09	-0.0011	0.0101	0.9129
rs10498846	6	67405337	T	C	0.473	0.00906	0.00178	6.62E-09	0.0044	0.0102	0.6689
rs1050847	16	87443734	C	T	0.495	0.0108	0.00179	1.67E-09	-0.0071	0.0099	0.4747
rs10905461	10	8803551	T	C	0.282	0.0107	0.00207	7.35E-09	0.0148	0.0114	0.1953
rs10945141	6	69470709	A	G	0.262	0.00825	0.00199	4.18E-08	0.0059	0.0112	0.5945
rs11057005	12	16748721	A	G	0.57	0.0104	0.0018	4.85E-09	-0.0019	0.0101	0.8545
rs11078713	17	7795972	A	G	0.546	0.0084	0.00182	2.23E-08	0.0292	0.0098	0.002832
rs11210196	1	73750567	C	T	0.506	0.0109	0.00177	1.15E-11	-0.0079	0.0097	0.4148
rs1154693	3	117804154	G	A	0.856	0.0145	0.00247	3.12E-11	0.005	0.0139	0.7216
rs1160685	4	94052854	G	C	0.478	0.00988	0.00178	7.20E-09	0.0038	0.0097	0.6912
rs11712680	3	75009019	A	C	0.826	0.0126	0.00225	3.51E-09	-0.0042	0.0134	0.7513
rs117143374	21	40555561	C	T	0.12	0.0125	0.00272	2.76E-08	0.0222	0.0147	0.1299
rs11721059	3	5725560	T	C	0.474	0.00895	0.00177	2.17E-08	-0.0218	0.01	0.02994
rs11768481	7	96629103	C	A	0.653	0.0103	0.00193	7.00E-10	-0.0221	0.0141	0.1167
rs117695734	11	111992273	A	G	0.9661	0.0269	0.00473	3.84E-10	-0.0177	0.0332	0.5946
rs11872397	18	72535282	G	A	0.748	0.012	0.00206	1.43E-09	-0.0065	0.0117	0.5818
rs11873164	18	42659922	C	T	0.865	0.0135	0.00248	1.31E-08	-0.0058	0.0138	0.6756

rs12112638	7	69735251	A	G	0.725	0.0108	0.00201	1.34E-09	0.0384	0.0144	0.007565
rs12186738	5	103816655	G	T	0.846	0.0133	0.00245	3.42E-11	0.0188	0.0142	0.184
rs12356821	10	104563808	C	G	0.14	0.0141	0.00256	6.27E-15	-0.0293	0.0143	0.04037
rs12441907	15	83922387	C	A	0.814	0.0127	0.00224	1.06E-10	-0.0026	0.0123	0.8338
rs12474587	2	162802993	T	G	0.404	0.0111	0.00179	1.25E-14	0.0096	0.0099	0.3314
rs12632110	3	50224225	A	G	0.353	0.0102	0.00187	4.78E-10	0.0144	0.01	0.1477
rs12727441	1	87915254	T	C	0.647	0.00908	0.00182	3.29E-08	-0.0031	0.0104	0.7668
rs13030994	2	146143090	A	G	0.485	0.0157	0.00176	3.56E-24	-0.0116	0.0095	0.2183
rs13109980	4	140886963	G	A	0.69	0.0103	0.00187	9.99E-11	0.0035	0.0107	0.7432
rs13261666	8	59814666	G	T	0.478	0.0113	0.00176	3.90E-14	-0.0225	0.01	0.0247
rs134529	22	28781758	T	C	0.651	0.00809	0.00182	4.85E-08	-0.02	0.01	0.04561
rs1385108	5	154839646	T	C	0.239	0.0121	0.00207	3.00E-09	-0.0093	0.0115	0.4176
rs1565735	8	27426077	T	A	0.788	0.0176	0.00227	3.42E-17	-0.0021	0.015	0.8913
rs160631	6	52895230	T	G	0.268	0.00938	0.00199	1.11E-08	0.0091	0.011	0.4093
rs17234745	9	11081182	T	C	0.847	0.0106	0.00256	1.53E-08	-0.0076	0.0142	0.5925
rs17417989	11	112713857	T	G	0.272	0.01	0.002	6.95E-09	0.0139	0.0117	0.2346
rs1899896	8	93201036	T	C	0.286	0.012	0.00194	1.04E-11	0.0101	0.0106	0.3417
rs1971318	12	121389500	T	C	0.141	0.0126	0.00244	7.06E-09	0.023	0.0132	0.08119
rs2046850	1	210304319	C	T	0.813	0.0108	0.00222	3.03E-08	5.0E-04	0.0126	0.9698
rs2107300	2	200937901	C	G	0.155	0.0134	0.00251	3.27E-08	0.0151	0.0136	0.2693
rs2186122	1	66470206	T	A	0.561	0.0125	0.00179	3.61E-13	-1.0E-04	0.0096	0.9902
rs2378662	9	86707289	A	G	0.556	0.00862	0.00178	4.16E-09	-0.0115	0.0098	0.24
rs240963	6	111644332	T	C	0.164	0.0175	0.00242	2.16E-17	-0.0187	0.0131	0.1559
rs266047	2	104088751	G	A	0.471	0.0134	0.00178	3.36E-16	-0.0095	0.01	0.3409

rs292071	4	28456089	C	T	0.244	0.0103	0.00198	4.08E-09	0.0144	0.0109	0.1839
rs3001723	1	44037685	A	G	0.321	0.0148	0.00192	8.12E-18	-0.0187	0.0104	0.07205
rs32006	5	106840779	G	A	0.852	0.0135	0.00244	2.67E-09	-0.0112	0.0144	0.4384
rs3904512	13	38357471	G	A	0.571	0.00867	0.00177	3.23E-09	-0.0082	0.0101	0.4157
rs4044321	5	166989513	A	G	0.358	0.0131	0.00185	6.08E-14	-0.0088	0.0105	0.3996
rs4236259	7	1708080	T	G	0.501	0.0114	0.0018	3.35E-12	6.0E-04	0.0105	0.9561
rs4378371	11	28608730	C	G	0.602	0.00899	0.00181	6.22E-09	0.0195	0.0103	0.05847
rs4440591	8	92773309	A	C	0.592	0.00796	0.00179	2.48E-08	0.0173	0.0097	0.07365
rs4523689	11	7950797	A	G	0.592	0.00757	0.00181	1.55E-08	0.0148	0.0095	0.1195
rs4543592	9	3014254	C	T	0.468	0.00887	0.00177	7.46E-10	-0.0152	0.0098	0.1227
rs4571506	5	87756918	C	T	0.508	0.0126	0.00177	1.09E-14	-0.0255	0.01	0.01097
rs4674993	2	226332033	A	G	0.793	0.0112	0.0022	1.32E-08	-0.0283	0.0126	0.02461
rs4759228	12	56508409	G	C	0.73	0.0101	0.00198	3.58E-08	-0.0088	0.0111	0.4272
rs4785836	16	65604652	T	C	0.602	0.00966	0.00182	2.26E-08	0.0052	0.0104	0.6135
rs56820925	20	54387374	C	T	0.653	0.0104	0.00184	1.73E-08	0.0168	0.0105	0.1103
rs57969627	2	145652706	T	G	0.294	0.00966	0.00191	7.69E-10	-0.0128	0.0106	0.2263
rs61774463	1	73065502	C	A	0.777	0.0101	0.00214	1.71E-08	-0.0032	0.0118	0.7843
rs6265	11	27679916	C	T	0.797	0.014	0.00224	3.77E-12	-0.0193	0.0117	0.09885
rs6433897	2	182034448	C	T	0.754	0.00999	0.00203	3.16E-08	0.0073	0.0112	0.5122
rs6460944	7	1876199	T	C	0.419	0.00969	0.00178	8.26E-10	0.0275	0.0101	0.006162
rs6508144	18	50026142	C	G	0.437	0.00883	0.0018	7.97E-09	-0.0263	0.0102	0.009489
rs6549058	3	85878104	T	C	0.621	0.0103	0.00183	1.67E-11	-0.007	0.0101	0.4882
rs6669839	1	50625979	T	C	0.204	0.0123	0.00218	3.36E-09	-9.0E-04	0.0126	0.9417
rs6728726	2	623976	C	T	0.829	0.0158	0.00235	6.73E-14	0.0123	0.0122	0.3137

rs6788098	3	85624131	A	T	0.377	0.0126	0.00183	1.91E-17	0.0156	0.0099	0.1128
rs6893752	5	60374912	A	G	0.234	0.0102	0.00201	3.25E-09	-5.0E-04	0.0115	0.9652
rs7224742	17	30657058	C	T	0.405	0.00808	0.00182	1.43E-08	-0.0024	0.0098	0.8065
rs7322872	13	100548329	C	T	0.218	0.0108	0.00219	3.58E-09	0.0054	0.0133	0.6843
rs7585579	2	60024857	G	C	0.505	0.00792	0.00179	1.88E-09	-0.0129	0.0101	0.1988
rs76214862	14	29500130	A	C	0.798	0.012	0.00227	3.99E-08	0.0341	0.0129	0.008127
rs76608582	19	4474725	C	A	0.9611	0.0273	0.00471	1.94E-09	-0.0123	0.0495	0.8044
rs7844990	8	65074004	C	T	0.401	0.0103	0.00182	3.23E-08	-0.01	0.0105	0.3425
rs7921378	10	63674885	G	C	0.537	0.0125	0.00178	8.26E-13	-0.0245	0.01	0.01482
rs7929518	11	85980958	G	A	0.765	0.0113	0.00212	1.56E-08	-0.002	0.0116	0.8652
rs7969559	12	69655167	A	G	0.312	0.00965	0.00197	7.31E-10	0.0209	0.0105	0.04737
rs846799	6	101270345	A	G	0.505	0.00917	0.00178	3.87E-09	-0.0087	0.0095	0.3584
rs9401770	6	98748008	A	G	0.273	0.0107	0.00198	3.47E-12	0.0247	0.0108	0.02269
rs9423279	10	125680419	C	G	0.359	0.00845	0.00197	3.21E-08	0.0208	0.0147	0.1565
rs9540729	13	66947124	A	T	0.499	0.00784	0.00176	3.82E-08	-1.0E-04	0.01	0.9898
rs962950	15	47685416	T	A	0.346	0.0118	0.00188	1.84E-12	-0.0199	0.0109	0.06808
rs993700	4	67825894	T	C	0.234	0.012	0.00213	1.53E-09	-0.0048	0.0117	0.6778

Supplementary Table 4. Variants associated with the *age at smoking initiation* (Age Init), their magnitude of effect and their strength of association with PD.

SNP	Chr	BP	EA	OA	EA freq	Age Init beta	SE Age Init beta	p-value Age Init association	PD beta	SE PD beta	p-value PD association
rs11780471	8	27344719	A	G	0.0644	0.0387	0.00809	1.73E-06	0.0053	0.0201	0.7918
rs11915747	3	85699040	G	C	0.354	0.0162	0.00399	4.63E-05	0.004	0.0098	0.6797
rs140485736	14	75360268	A	G	0.0131	0.0599	0.0158	0.000149	-0.1212	0.1011	0.2307
rs319748	17	31554533	G	A	0.289	0.0107	0.00418	0.0106	0.0071	0.0113	0.5336
rs3768886	2	225450161	C	G	0.328	0.0163	0.00408	6.46E-05	-0.0119	0.0107	0.2682
rs624833	4	2881256	G	T	0.309	0.0136	0.00414	0.00107	0.0472	0.0102	3.66E-06
rs7599208	2	63622470	C	T	0.442	0.0193	0.00383	4.84E-07	-0.0167	0.009	0.09187

Supplementary Table S5. Variants associated with *smoking heaviness (cigarettes per day)*, their magnitude of effect on CPD and the strength of association with PD.

SNP	Chr	BP	EA	OA	EA freq	CPD beta	SE CPD beta	p-value CPD association	PD beta	SE PD beta	p-value PD association
rs116980959	19	41267073	T	C	0.99041	0.0682	0.0274	0.0129	-0.0062	0.0607	0.918
rs11725618	4	67053769	C	T	0.287	0.0167	0.00415	5.32E-05	-0.0102	0.0121	0.4001
rs139648962	15	79067452	G	A	0.967	0.0554	0.0101	4.02E-08	-0.0034	0.0419	0.935
rs147893869	19	41321074	C	G	0.9775	0.0821	0.0119	4.55E-12	0.0719	0.0388	0.06366
rs2072659	1	154548521	C	G	0.895	0.0305	0.00637	1.71E-06	0.0423	0.019	0.02591
rs2084533	3	16872929	T	C	0.319	0.018	0.00399	6.37E-06	0.0102	0.0107	0.3433
rs215600	7	32333642	G	A	0.36	0.0249	0.00392	2.06E-10	-0.0068	0.0099	0.4967
rs2273500	20	61986949	C	T	0.159	0.0345	0.00532	8.58E-11	-0.001	0.0158	0.9478
rs2386571	16	52074123	A	C	0.43	0.0227	0.00379	2.05E-09	0.024	0.0096	0.0126

rs3025383	9	136502369	T	C	0.82	0.0319	0.00471	1.19E-11	-0.0315	0.0122	0.009783
rs56113850	19	41353107	C	T	0.568	0.0402	0.00383	8.58E-26	0.0207	0.0117	0.07728
rs58379124	8	42579203	C	T	0.748	0.0336	0.00439	2.06E-14	-0.0114	0.0114	0.3179
rs6508965	19	41517688	T	C	0.381	0.0134	0.00395	0.000753	0.0099	0.0103	0.3351
rs72738727	15	78749235	C	T	0.9819	0.0534	0.015	0.00037	0.0086	0.0511	0.867
rs73229090	8	27442127	A	C	0.113	0.0255	0.00602	2.21E-05	-0.0008	0.015	0.9577
rs7431710	3	48935583	G	A	0.356	0.0176	0.00396	9.26E-06	-0.0562	0.0099	1.28E-08
rs75494138	11	46465361	T	C	0.0618	0.0388	0.00725	8.96E-08	0.0164	0.0187	0.3801
rs75596189	9	136468701	T	C	0.0954	0.0311	0.00595	1.65E-07	-0.0367	0.0156	0.0189
rs76209839	19	41233815	C	T	0.9697	0.0469	0.0103	5.22E-06	0.0432	0.0396	0.2756
rs790564	8	64604218	A	C	0.281	0.0188	0.00423	8.79E-06	0.0357	0.0112	0.001467
rs7928017	11	113448762	C	A	0.587	0.0155	0.00378	4.36E-05	-0.0049	0.0103	0.6311
rs7951365	11	16377044	C	T	0.306	0.0195	0.00408	1.81E-06	-0.0062	0.0116	0.5944
rs8029039	15	79100871	C	G	0.759	0.0326	0.00435	7.43E-14	0.0114	0.0116	0.3266
rs8034191	15	78806023	C	T	0.328	0.0904	0.00404	1.10E-110	0.0011	0.0098	0.9085
rs895330	19	4060707	C	G	0.794	0.184	0.00476	1.09E-04	-0.0457	0.0128	3.55e-04
